# *In situ* observation of reactive oxygen species forming on oxygen-evolving iridium surfaces†
†Electronic supplementary information (ESI) available. See DOI: 10.1039/c6sc04622c



**DOI:** 10.1039/c6sc04622c

**Published:** 2016-12-01

**Authors:** Verena Pfeifer, Travis E. Jones, Juan J. Velasco Vélez, Rosa Arrigo, Simone Piccinin, Michael Hävecker, Axel Knop-Gericke, Robert Schlögl

**Affiliations:** a Department of Inorganic Chemistry , Fritz-Haber-Institut der Max-Planck-Gesellschaft , Faradayweg 4-6 , 14195 , Berlin , Germany . Email: trjones@fhi-berlin.mpg.de; b Catalysis for Energy , Group EM-GKAT , Helmholtz-Zentrum Berlin für Materialien und Energie GmbH , Elektronenspeicherring BESSY II , Albert-Einstein-Str. 15 , 12489 , Berlin , Germany; c Department of Heterogeneous Reactions , Max-Planck-Institut für Chemische Energiekonversion , Stiftstr. 34-36 , 45470 , Mülheim a. d. Ruhr , Germany; d Diamond Light Source Ltd. , Harwell Science & Innovation Campus , Didcot , Oxfordshire OX 11 0DE , UK . Email: rosa.arrigo@diamond.ac.uk; e Consiglio Nazionale delle Ricerche – Istituto Officina dei Materiali , c/o SISSA , Via Bonomea 265 , Trieste , 34136 , Italy

## Abstract

Water splitting performed in acidic media relies on the exceptional performance of iridium-based materials to catalyze the oxygen evolution reaction (OER). In the present work, we use *in situ* X-ray photoemission and absorption spectroscopy to resolve the long-standing debate about surface species present in iridium-based catalysts during the OER. We find that the surface of an initially metallic iridium model electrode converts into a mixed-valent, conductive iridium oxide matrix during the OER, which contains O^II–^ and electrophilic O^I–^ species. We observe a positive correlation between the O^I–^ concentration and the evolved oxygen, suggesting that these electrophilic oxygen sites may be involved in catalyzing the OER. We can understand this observation by analogy with photosystem II; their electrophilicity renders the O^I–^ species active in O–O bond formation, *i.e.* the likely potential- and rate-determining step of the OER. The ability of amorphous iridium oxyhydroxides to easily host such reactive, electrophilic species can explain their superior performance when compared to plain iridium metal or crystalline rutile-type IrO_2_.

## Abstract 2

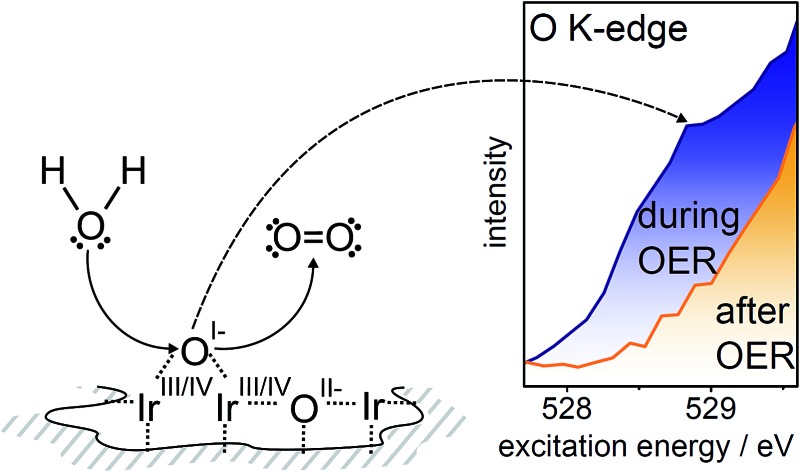
In situ XAS measurements reveal that electron-deficient oxygen species form during OER on IrOx and correlate with catalytic activity.

## 1 Introduction

In addition to needing renewable energy technologies, an economy based on sustainable resources requires efficient energy storage options. Water electrolysis presents an attractive solution to the latter requirement; it stores excess energy from renewable sources in chemical bonds.^1,2^ To be effective, electrolyzers must be capable of adapting to the varying power inputs of intermittent renewable sources like wind and solar power. While electrolyzers based on proton exchange membranes (PEM) meet this demand, they require a corrosive acidic working environment. Therefore, current research on water electrolysis is driven by the need for efficient, stable, and cost-effective electrocatalysts for the sluggish oxygen evolution reaction (OER) in acidic media.^3^

While iridium oxide is rare and precious, it remains the state-of-the-art OER catalyst in acidic media owing to its high activity, low overpotential, and good stability.^4^ To uncover the reason for iridium oxide's increased activity, surface scientists have long examined its electronic structure. By combining electrochemical measurements with *ex situ* X-ray photoemission spectroscopy (XPS), they provided chemical information about oxidation states and elemental abundance present after emersion at various potentials.^5,6^ The major drawback of such *ex situ* experiments is that the active state of the electrode cannot be observed under reaction conditions. Furthermore, since the active iridium surface state is likely a hydrated and hydroxylated amorphous form of iridium oxide,^5,7,8^ the electrode's surface morphology and composition likely change when emersed from the electrolyte and brought into a UHV environment.^5,9^ Thus, the active sites may be lost or modified, and since Fierro *et al.*'s^10^ isotope-labeling experiments showed that the reactive oxygen species contained in the iridium-oxide matrix are directly involved in the catalytic OER cycle, an *in situ* investigation elucidating the chemical nature of these reactive species is highly desirable.

Significant effort has been invested in the development of *in situ* methodology to assess the active state of electrode materials under working conditions. Owing to these efforts, it is now possible to drive electrochemical reactions and simultaneously record XPS^11–13^ and X-ray absorption spectroscopy (XAS)^14,15^ to monitor the electronic structure of oxygen-evolving surfaces *in situ*. Nevertheless, the interpretation of XPS and XAS measurements of iridium oxides, especially *in situ*, remains challenging. While the literature agrees that hydrated and hydroxylated amorphous forms of iridium oxide with mixed iridium oxidation states have intrinsically higher OER activities than pristine iridium metal and crystalline rutile-type IrO_2_,^7,12,15,16^ dissent remains about which types of iridium surface species are present during the catalytic OER cycle.^12,15^

The difficulties in pinpointing iridium oxidation states partly originate from the lack of well-defined oxidic iridium reference materials other than the tetravalent Ir in rutile-type IrO_2_.^17^ Drawing parallels with iridium species with different oxidation states present in non-conductive, non-oxidic reference materials is hampered by the fact that these species often have only small^18,19^ or reverse^20^ shifts in excitation or binding energy in NEXAFS and XPS and usually overlap to a large extent. Therefore, while *in situ* XPS investigations were interpreted to show the presence of iridium species with oxidation states of IV and V during the OER,^12^ *in situ* XAS measurements were deconvoluted into contributions of Ir^III^ and Ir^V^.^15^

In contrast to the difficult identification of the iridium species present, oxygen species (formally O^I–^ and O^II–^) contained in highly OER-active X-ray amorphous iridium oxide structures show clear fingerprints in the near-edge X-ray absorption fine structure (NEXAFS) of the O K-edge.^8,16,21^ These fingerprint features are highly sensitive to changes in the electronic structure of iridium oxides.^22^ Hence, the identification of oxygen species in iridium oxide structures presents a key to interpret features in the electronic structure of active iridium oxide surfaces and to shed light on iridium oxide's remarkable activity. For the oxygen species contained in the highly active, amorphous iridium oxide catalysts, for example, the formally O^I–^ species has recently been identified as a highly reactive, electrophilic oxygen.^22^

Such electrophiles are prone to nucleophilic attack. This susceptibility to attack by water or OH likely makes O^I–^ active in O–O bond formation, which is often described as both the potential-determining^23^ and rate-limiting^24^ step of the OER. Since we know the electronic structure fingerprints of electrophilic O^I–^ species,^16,21^ we can test if they are indeed forming in oxygen-evolving iridium surfaces by *in situ* NEXAFS and XPS.

On this account, we make use of a PEM-based *in situ* technique^11^ for investigating gas-phase water electrolysis. This technique enables us to record XPS and NEXAFS while a model iridium electrode evolves oxygen. An advantage of our approach is that, by keeping the oxygen chemical potential in our system low, we are able to investigate the initial stages of oxide formation on iridium surfaces during the OER. To ensure these low oxygen chemical potentials yield relevant results, we first demonstrate at high overpotentials how iridium is oxidized during the OER and identify the nature of the iridium and oxygen species formed during the reaction. In the second step, we perform a controlled experiment near the onset of the OER activity of iridium to identify a correlation between the amount of evolved oxygen and the concentration of oxygen species present on oxygen-evolving iridium surfaces.

## 2 Experimental

The discrepancies between electrochemical measurements in electrolyte and surface-sensitive spectroscopic investigations in UHV have been bridged in recent years by setups combining both techniques.^11–13^ The present study uses an *in situ* cell for investigating gas-phase water electrolysis described in detail by Arrigo *et al.* ^11^ In this cell concept (see Fig. S1 in ESI†), a Nafion® proton exchange membrane (PEM) is used to separate a continuous flow of liquid water/electrolyte from the evacuated measurement chamber of the near-ambient-pressure XPS (NAP-XPS) endstation of the ISISS beam line^25^ at the synchrotron facility BESSY II/HZB, Berlin, Germany. The cell concept is described in detail in the ESI.†

In brief, water diffuses through the desiccation cracks of the ≈10 nm thick, sputter-deposited electrodes and the PEM due to the pressure difference between the liquid on one side of the membrane and the evacuated measurement chamber on the other (see Fig. S2–S4 in ESI†). The water both hydrates the PEM, ensuring good ion conductivity, and delivers the reactant molecules to the working electrode with the resultant equilibrium pressure reaching 0.1–10 Pa. By connecting the working (Ir) and the counter (Pt) electrodes to an external potentiostat, OER-relevant potentials can be applied. A quadrupole mass spectrometer (QMS) monitors the gas composition on-line to test if oxygen is evolving from the iridium surface.

With this setup, we are able to record XPS and NEXAFS of the X-ray-exposed model iridium working electrode while the OER proceeds. To work under more controlled conditions, in the second part of our study, we equipped the cell with a Ag/AgCl reference electrode (see Fig. S1 in ESI†).

## 3 Results and discussion

**Fig. 1:**
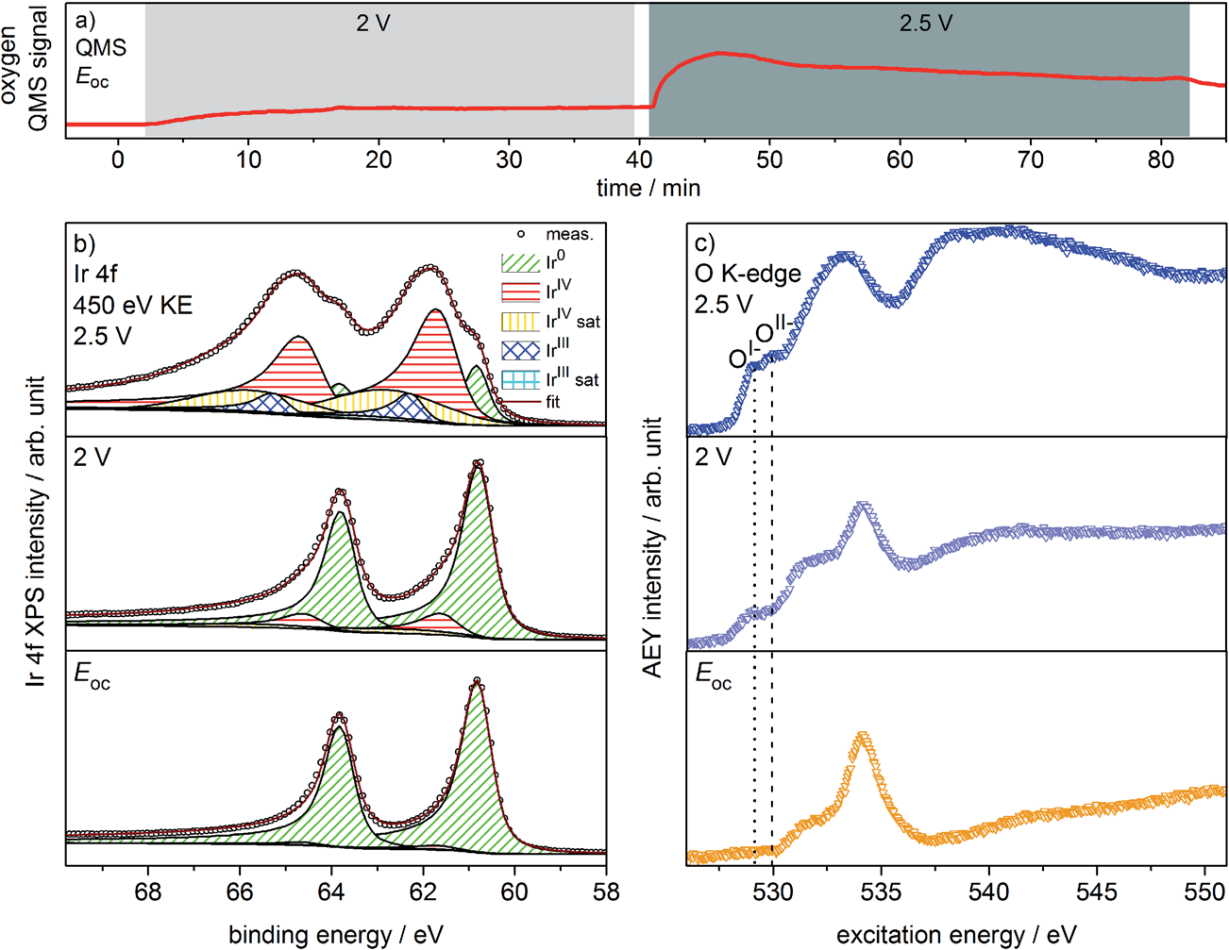
(a) Oxygen QMS trace, (b) Ir 4f spectra and (c) O K-edges of Ir-coated PEM (120 s Ir sputtered) recorded in the two-electrode cell with the indicated potentials applied (*p* = 5 Pa, H_2_O).

Oxygen production on the iridium working electrode is a requirement for our PEM-based approach to deliver relevant observations. Our on-line QMS confirms this OER-active condition of the electrode (see Fig. 1a). When switching the potential from *E*_oc_ (open circuit potential) to 2 V, we observe an increase in the oxygen QMS trace. When further increasing the potential to 2.5 V, we detect an additional increase in the oxygen QMS trace. As expected, higher potentials lead to a higher OER activity. By contrast, if we introduce a PEM without Ir coating into the cell, the oxygen QMS trace does not increase when we apply OER-relevant potentials (see Fig. S7 in ESI†). Hence, the oxygen detected in the case of the Ir-coated sample must be produced at the Ir working electrode.

Ir 4f and O K-edge spectra (probing depths of ≈2 nm and 2–3 nm, respectively) were collected at each of the potentials applied to the Ir working electrode of the two-electrode system to monitor changes in the present iridium and oxygen species (see Fig. 1b and c). A deconvolution of the Ir 4f spectra, *i.e.* a speciation of the Ir species, can be done employing the fit model for iridium oxides introduced in our previous work.^16,21^ Good agreement is obtained between the recorded spectra and the fit envelope for all core level spectra (for fit parameters see Table S1 in ESI†).

At *E*_oc_, the Ir 4f spectrum (see Fig. 1b) of a pristine, as-deposited iridium film is dominated by the contribution of metallic Ir at 60.8 eV, which has an asymmetric line shape typical for metallic conductors.^26^ In addition, a minute oxidic component is present at higher binding energy, likely due to surface oxidation of the sputtered Ir nanoparticles. Increasing the potential to 2 V to start the OER produces only subtle changes in the Ir 4f spectrum. The metallic contribution remains nearly unchanged and the oxidic component grows slightly. Further increasing the potential to 2.5 V leads to pronounced changes, with substantially more intensity emerging at higher binding energies in a broad feature.

The Ir 4f spectrum recorded during the OER at 2.5 V consists of three major contributions. First, there is a residual of the initially metallic Ir at 60.8 eV binding energy. Second, the largest contribution centered at 61.7 eV originates from iridium in the formal oxidation state IV (as in the well-defined reference rutile-type IrO_2_). This peak has an asymmetric line shape and appears in combination with a satellite at 1 eV higher binding energy.^16,21^ Finally, there is an additional component at 62.3 eV, *i.e.* at higher binding energy than Ir^IV^, not found in rutile-type IrO_2_. The appearance of this higher binding energy iridium oxide feature during OER was also observed by Casalongue *et al.* ^12^ Based on the shift to higher binding energy, they intuitively assigned this feature to Ir^V^. Nevertheless, in our previous work we combined XPS with theoretical calculations and concluded that Ir^III^ species can exhibit a reverse binding energy shift and that these species are also expected to be found at higher binding energies than Ir^IV^, namely 62.3 eV.^16,21^ Such Ir^III^ species are present in amorphous, highly OER-active iridium oxyhydroxides. In these materials, the presence of both Ir^III^ and formally O^I–^ has been identified. Hence, an identification of the oxygen species formed during OER may help us shed light on the nature of these additional Ir species present during the OER at 2.5 V.

We can assess the nature of the oxygen species present in the near-surface region of the catalyst by inspecting the O K-edges collected at the different potentials (see Fig. 1c). In the O K-edge spectrum recorded at *E*_oc_, we mainly see contributions of carbonaceous contamination of the surface (532–535 eV) and possibly sulfonic or sulfate groups of the PEM (>537 eV) (see Fig. S8 in ESI†). In accordance with the Ir 4f spectrum collected at *E*_oc_, we observe no clear contribution of iridium oxide species in the corresponding O K-edge. By contrast, at 2 V where we only registered a small contribution of an oxidic component to the Ir 4f spectrum, we see two clearly visible contributions appearing in the low excitation energy region of the O K-edge. The excitation energy values of these two contributions, namely 529 eV and 530 eV, match the main resonances of formally O^I–^ and O^II–^ species identified in the amorphous iridium oxyhydroxide reference material exactly.^16,21^ When further increasing the potential to 2.5 V, the O^I–^ and O^II–^ contributions to the O K-edge grow in intensity.

While we can imagine O–O groups to be present on the iridium surface as intermediates of the OER, our calculations of the spectroscopic fingerprints of superoxo and peroxo species show that such species cannot account for the observed low excitation energy feature at 529 eV (see Fig. S31 in ESI†). Since the spectral features seen in the iridium surface oxidized during OER coincide with those observed in the amorphous Ir^III/IV^ oxyhydroxide reference, we suggest that these materials are of a similar nature. We tentatively identify the iridium species present on oxygen-evolving iridium surfaces as Ir^III^ and Ir^IV^. Further, we confirm the formation of O^I–^ and O^II–^ species during the OER over iridium. Hence, we have witnessed *in situ* that, during the OER, electrophilic O^I–^ species form in an amorphous, mixed-valent iridium phase.

**Fig. 2:**
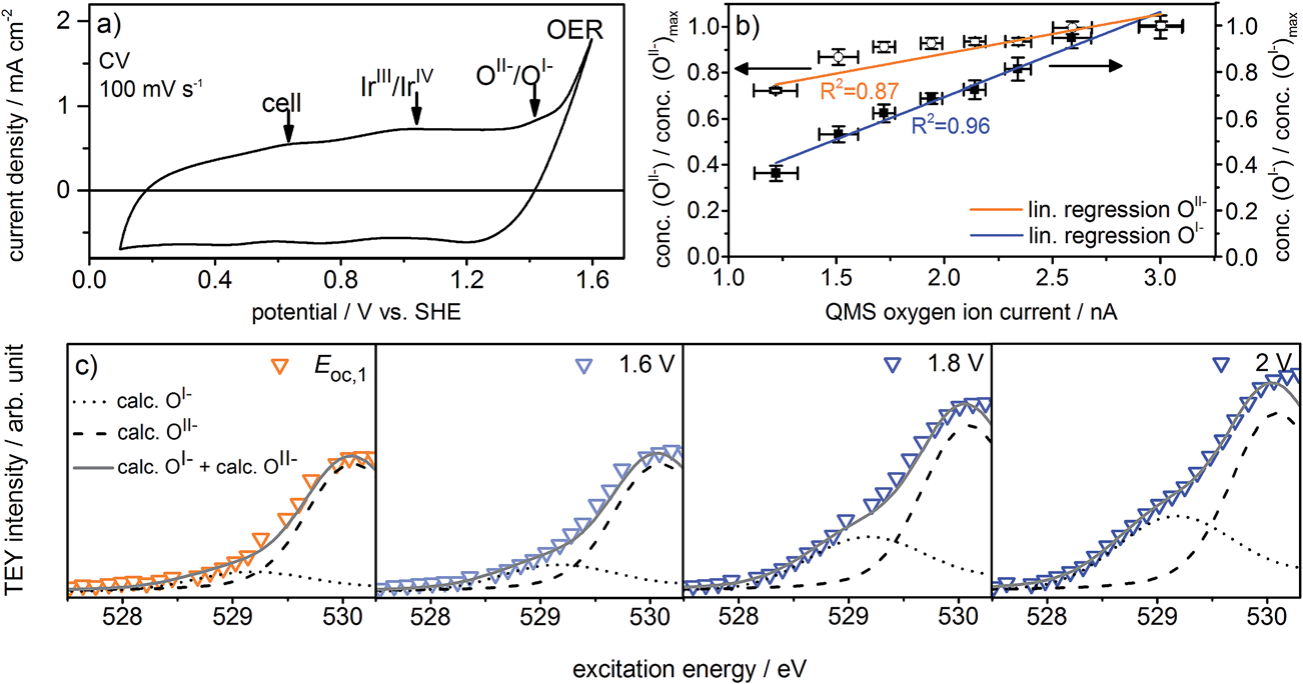
(a) Cyclic voltammogram, (b) normalized O^I–^ and O^II–^ concentrations over QMS oxygen ion current, and (c) zoomed and fitted low excitation energy regions of O K-edges recorded in the three-electrode cell (indicated potentials *vs.* SHE, ring current = 60 mA, *p* = 0.3 Pa, 0.1 M H_2_SO_4_).

The observed formation of O^I–^ species during the OER not only strengthens our previous suggestion that such electrophilic O^I–^ species may be crucial for the OER reactivity^16,21,22^ but may also explain the long-standing question about the origin of the oxidation signal observed at ≈1.4 V *vs.* SHE in the CV of iridium (see Fig. 2a). Whereas in the past this signal was often assigned to further oxidation of the metal center, *i.e.* the transition of Ir^IV^ to Ir^V^,^5,27,28^ we suggest that it reflects the oxidation of oxygen from O^II–^, contained in the IrO_*x*_ matrix in form of adsorbed OH groups, to O^I–^:

1 IrO_*x*_O^II–^H ⇌ IrO_*x*_O^I–^ + H^+^ + e^–^

To test this assertion, we used density functional theory (see ESI†) to compute the potential at which O^I–^ forms from surface OH groups, the latter of which give resonances at 532 eV in the O K-edge (see Fig. S28 in ESI†). The computed value of 1.2–1.3 V supports the hypothesis that O^I–^ formation accounts for the ≈1.4 V oxidation signal observed experimentally. While such a redox-active, non-innocent ligand^29,30^ may at first glance contrast with common intuition, the diffuse nature of the Ir 5d orbitals and the metal's high oxidation state (IV) in IrO_2_ suggest it is energetically favorable for the oxide to accommodate holes in the O 2p states rather than further oxidize Ir^IV^ to Ir^V^.^31^

With these experiments, we have illustrated the OER-active surface state of an iridium electrode likely consists of an Ir^III/IV^ matrix with oxygen species in the formal oxidation states I– and II–. A comparison of our observations from the recorded Ir 4f spectra and O K-edges highlights that although we only observe subtle changes in the Ir 4f spectrum at 2 V during the OER, the appearance of iridium oxygen compounds is clearly mirrored by the presence of O^I–^ and O^II–^ species in the O K-edge. Hence, the O K-edge is more sensitive to changes in the electronic structure of iridium oxides than the Ir 4f core line. Therefore, we use the O K-edge as basis for the subsequent investigation to determine which species are present near the onset of the OER and how they correlate with activity.

What remains to be clarified is how far the presence of the observed oxygen species is related to iridium's OER activity. For this aim, the reaction needs to be driven under exact potential control at moderate overpotentials. To conduct such experiments near the onset of iridium's OER activity, we upgraded the cell into a three-electrode system by adding a Ag/AgCl reference electrode (Fig. S1 in ESI†), which ensures that the potential applied to the working electrode has exactly the desired value.

Fig. 2a shows the redesigned device works under relevant conditions. The CV of a sputter-deposited Ir electrode displays the characteristic oxidation waves at 1 V *vs.* SHE (commonly attributed to an oxidation of Ir^III^ to Ir^IV^ ^27,28^) and 1.4 V *vs.* SHE (oxidation of oxygen from O^II–^ to O^I–^, see the preceding discussion) and the OER onset at 1.5 V *vs.* SHE. The additional feature visible at 0.6 V *vs.* SHE originates from the cell itself (see Fig. S6 in ESI†). After a series of 35 CVs to precondition the iridium electrode surface, the potential applied to the working electrode was stepwise increased by 0.05 V starting from 1.6 V *vs.* SHE. Fig. S18 in ESI† shows the expected concomitant increase of both the current density and the oxygen concentration in the gas phase. A plot of oxygen concentration over current density shows their linear relationship (see Fig. S18 in ESI†) excluding that, within the measured potential region, the ratio between currents due to simple corrosion and oxygen production changes. Furthermore, an estimation of the amount of present Ir atoms (≈10^16^) and the overall number of electrons passed across the electrode (≈10^19^) excludes a major contribution to the measured current by simple Ir dissolution/corrosion. By these potential steps, we have created iridium oxide surfaces that evolve different amounts of oxygen. To see whether this increase in oxygen production also shows a correlation with the concentration of the present oxygen species, we recorded the O K-edge at each applied potential (see Fig. S19 in ESI†).

Fig. 2c shows the low excitation energy region of the O K-edge at successively applied potentials. We deconvoluted these regions using the calculated spectra for the O^I–^ and O^II–^ species^16,21^ to quantify their respective contributions to the overall signal. We used the resulting fits to determine the dependence of the O^I–^ and O^II–^ concentrations on the current density and oxygen QMS ion current (see Fig. 2b, S16 and S21 in ESI†). While we observe a linear correlation between the oxygen evolution activity and the O^I–^ concentration (*R* ^2^ = 0.96), we see only a weak correlation with the O^II–^ concentration (*R* ^2^ = 0.87). Hence, the concentration of O^I–^ seems to be tied to the magnitude of the OER reactivity of iridium catalysts whereas the concentration of O^II–^ does not.

**Fig. 3:**
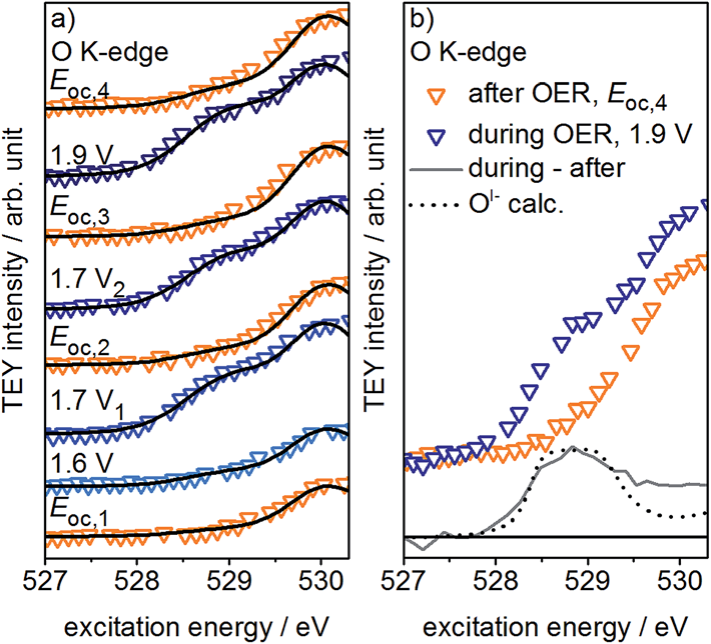
(a) Low excitation energy region of O K-edges of Ir-coated PEM (60 s Ir sputtered), consecutively recorded (bottom to top) in the three-electrode cell with the indicated potentials *vs.* SHE applied and (b) difference spectrum of two consecutively recorded O K-edges and comparison with calculated O^I–^ spectrum (ring current = 13 mA, *p* = 0.45 Pa, 0.1 M H_2_SO_4_).

If, as we hypothesize, the O^I–^ species in these experiments are stabilized by the applied potential, their concentration should decrease once the potential is reduced below 1.4 V *vs.* SHE. Aiming to relate the presence of these reactive species to the presence of an applied, OER-relevant potential, we alternatively turned on and off the potential using another fresh sample (see Fig. S22–S24 in ESI†). In the recorded and fitted spectra, we clearly observe that turning on and off the potential also reversibly turns on and off the major contribution to the intensity of the O^I–^ species (see Fig. 3a). The difference of the spectra recorded during and after OER (labeled during-after in Fig. 3b) yields close agreement with the calculated O^I–^ spectrum.

The disappearance of the O^I–^ species once the potential is switched off is likely due to their protonation and shift to higher excitation energies (≈532 eV, see Fig. S28 in ESI†). Unfortunately, owing to the high background signal in this excitation energy region, we are not able to verify such an increase in intensity. Nevertheless, such a protonation and subsequent increase in concentration of hydroxyl groups is in agreement with Reier *et al.*'s observation that post reaction, highly active OER catalysts have high concentrations of OH-groups at the surface.^8^ The small residual of O^I–^ still present in our experiments after the potential is turned off is likely due to subsurface sites that are not accessible for protonation. As the thickness of the amorphous oxide layer increases, we would then expect a concomitant increase in the amount of residual O^I–^, in agreement with its *ex situ* presence in highly active amorphous iridium oxyhydroxide powders.^16,21^ However, the fraction of residual O^I–^ in our thin oxide films is minute when compared to the large O^I–^ signal in such bulk amorphous powder catalysts with signal contributions from deeper, possibly inaccessible subsurface O^I–^ species. Hence, while in the present experiment the majority of O^I–^ is likely protonated and forms OH-groups once the potential is switched off, many of the O^I–^ hosted by the bulk oxyhydroxides seem to be unprotonated.

If we assume that the increase in O^I–^ concentration with increasing oxygen evolution activity is not a mere side reaction, we can combine our *in situ* observations to address the original hypothesis that the electrophilic O^I–^ species participates in O–O bond formation during the OER on iridium. This idea stems from a comparison to photosystem II (PS II). Although in this heavily studied biological system for oxygen generation from water the O–O bond formation process has not yet been finally resolved, theory and experiment strongly agree on the involvement of an electron-deficient oxygen species, either electrophilic oxygen or an oxyl radical, in O–O bond formation.^32–37^ Also for other water oxidation catalyst systems the presence of such electron-deficient intermediates has been reported based on vibrational measurements.^38–41^ While, so far, we have not obtained direct experimental evidence for the formation of radicals in the iridium oxide matrix, our previous study^22^ demonstrated the O^I–^ species, shown in the present study to also form in oxygen-evolving iridium surfaces, to be strong electrophiles. In a mechanism proposed for PS II, which involves such electrophilic oxygen species, an oxygen contained in the Mn water oxidation complex (WOC) transforms into an electrophile (O*) that is subsequently attacked by nucleophilic (bound) water or hydroxides to form the O–O bond.^35–37^ In a simplified form, this part of the OER process in PS II can be written as:

2 WOC–O* + H_2_O → WOC–O^I–^–O^I–^–H + H^+^ + e^–^ → WOC + O_2_ + 2H^+^ + 3e^–^

Under the assumption that a similar mechanism with a ligand-centered oxidation prior to O–O bond formation applies to the OER over iridium oxides, we may write for the reaction between water and the electrophilic O^I–^ species observed on iridium oxides during the OER:

3 IrO_*x*_O^I–^ + H_2_O → IrO_*x*_O^I–^–O^I–^–H + H^+^ + e^–^ → IrO_*x*_ + O_2_ + 2H^+^ + 3e^–^

The electrophilic nature of the O^I–^ makes this nucleophilic attack of water possible, whereas the additional charge on O^II–^ makes that species less susceptible to such an attack. After the evolution of oxygen, the catalytic cycle can be closed by regenerating IrO_*x*_O^I–^ from IrO_*x*_ and water under the influence of the applied potential. And while we assumed a nucleophilic mechanism in reaction (3) due to suggestions from theory^23^ and ultra-fast infra-red measurements^42^ that an OOH intermediate is formed during OER on iridium, we could have formulated the reaction by assuming the O^I–^ observed in this work has significant radical character. The crucial point is that electron-deficient oxygen is required in either mechanism. Thus, the high activity observed for amorphous iridium oxyhydroxide powder catalysts is then apparent from their ability to form large amounts of highly reactive, electron-deficient O^I–^ species.^16,21,22^ In contrast to these highly active IrO_*x*_ powders, rutile-type IrO_2_ does not tend to form a high concentration of O^I–^ and is significantly less active in the OER.^16,21^ Thus, a catalyst's propensity to accommodate electrophilic O^I–^ species appears to be essential in its ability to catalyze O–O bond formation.

## 4 Conclusion

In conclusion, using *in situ* X-ray photoemission and absorption spectroscopies, we have demonstrated for the first time that reactive electrophilic O^I–^ oxygen species form in a mixed-valent iridium^III/IV^ matrix during the OER. The observed formation of these O^I–^ species implies that iridium oxide contains redox-active, non-innocent ligands accounting for the redox chemistry of the material. We further found the O^I–^ concentration to increase with the measured oxygen evolution activity and to virtually disappear from our thin oxyhydroxide films in the absence of an applied potential. Both observations agree with our hypotheses that electrophilic O^I–^ species are active in catalyzing the OER on iridium oxides and that enhanced OER performance of iridium oxyhydroxides can be achieved by the accommodation of large amounts of reactive oxygen species serving as precursor sites for the O–O bond formation. In the quest for less expensive materials to catalyze the OER, these findings can guide the way for the design of new high-performance catalysts comprising such reactive oxygen species.
