# Supplementary material for: In situ observation of reactive oxygen species forming on oxygen-evolving iridium surfaces
Source: Chem Sci. 2016 Dec 1;8(3):2143–9. doi: 10.1039/c6sc04622c (PMC5407268; doi:10.1039/c6sc04622c)
Supplement: Supplementary file 1 [file SC-008-C6SC04622C-s001.pdf]

Electronic Supplementary Material (ESI) for Chemical Science.
This journal is © The Royal Society of Chemistry 2016

Supplementary Information to

In situ observation of reactive oxygen species forming on oxygen-evolving iridium surfaces

Verena Pfeifer,[ab] Travis E. Jones,[a*] Juan J. Velasco Vélez,[ac] Rosa Arrigo,[d*] Simone Piccinin,[e] Michael Hävecker,[ac] Axel Knop-Gericke[a] and Robert Schlögl[ac]

a *Department of Inorganic Chemistry, Fritz-Haber-Institut der Max-Planck-Gesellschaft, Faradayweg 4-6, Berlin, 14195, Germany. E-mail: trjones@fhi-berlin.mpg.de*

b *Catalysis for Energy, Group EM-GKAT, Helmholtz-Zentrum Berlin für Materialien und Energie GmbH, Elektronenspeicherring BESSY II, Albert-Einstein-Str. 15, Berlin, 12489, Germany*

c *Department of Heterogeneous Reactions, Max-Planck-Institut für Chemische Energiekonversion, Stiftstr. 34-36, Mülheim a. d. Ruhr, 45470, Germany*

d *Diamond Light Source Ltd., Harwell Science & Innovation Campus, Didcot, Oxfordshire OX 11 0DE, UK. E-mail: rosa.arrigo@diamond.ac.uk*

e *Consiglio Nazionale delle Ricerche - Istituto Officina dei Materiali, c/o SISSA, Via Bonomea 265, Trieste, 34136, Italy*

Contents

1 ***In situ* X-ray photoemission/absorption spectroscopy setup** **3**

2 **Proton exchange membrane-based *in situ* cells** **4**

3 **Control investigation of uncoated PEM** **8**

4 **Ir 4f fit parameters** **12**

5 ***In situ* investigation near the onset of iridium's OER activity** **13**

6 **Calculation details** **22**

1 *In situ* X-ray photoemission/absorption spectroscopy setup

All *in situ* photoemission and absorption measurements were collected with the near-ambient-pressure X-ray photoemission spectroscopy (NAP-XPS) system at the ISISS (Innovative station for *in situ* spectroscopy) beam line[1] located at the synchrotron radiation facility BESSY II/HZB (Berlin, Germany). In contrast to conventional UHV-based XPS systems, in this setup spectra can be collected at gas-phase pressures of up to several hundreds of pascals due to a sophisticated differential pumping and electrostatic lens system.[2] In the present experiments, the pressure was adjusted between 0.1 Pa and 10 Pa depending on the measurement requirement as will be stated in more detail in the respective sections. All measurements were collected at room temperature.

For all XPS measurements, a pass energy (PE) of 20 eV and an exit slit setting of the beam line of 111 μm were used, which led to an approximate resolution of the Ir 4f core line of 0.5 eV at 450 eV kinetic energy (KE) of the photoelectrons. The corresponding inelastic mean free path of the photoelectrons is ≈0.7 nm according to the model of Tanuma *et al.*[3] A binding energy calibration of the spectra was realized by measuring the Fermi edge after each core level scan.

For all near-edge X-ray absorption fine structure (NEXAFS) measurements, the photon energy was varied between 525 eV and 552 eV by continuously moving the monochromator. Both the Auger and total electron yield (AEY and TEY) were registered. The AEY was measured with the electron spectrometer. To partly suppress the contribution of gas-phase oxygen and water,[4] the KE of the collected electrons was set to 385 eV with a PE of 50 eV. The TEY was collected via a Faraday cup via the first aperture of the differential pumping system with an applied accelerating voltage. While AEY is slightly more surface sensitive than TEY (probing depths of 2 nm - 3 nm vs. 5 nm - 10 nm),[5] TEY usually provides better signal-to-noise ratios which becomes important when measuring low intensities. When comparing NEXAFS spectra of a series of measurements, the spectra were normalized to 0 in the pre-edge region between 523 eV and 527 eV and a linear fit function of the same region was used to subtract the slightly linearly increasing background of the spectra.

The NAP-XPS system is equipped with an on-line quadrupole mass spectrometer (QMS, Prisma, Pfeiffer Vacuum, Inc., Germany). The QMS was used to continuously record the traces of H_2O, H_2, O_2, CO_2, and their fragments during the measurements.
The measurements reported here were collected during different operation modes of the synchrotron. When denoted with top-up mode, the storage ring current was held constant at 300 mA by continuous injections. To reduce beam damage of the sample, measurements were also collected during a low-alpha operation mode of the synchrotron, in which the

storage ring current was in decay mode starting from 100 mA or 15 mA. The used mode and storage ring current will be denoted in the respective paragraphs.

2 Proton exchange membrane-based *in situ* cells

In the present work, the design of an *in situ* cell described by Arrigo *et al.*[6] based on the water permeability of a proton exchange membrane (PEM) was further developed. Due to the modular approach of the ISISS endstation, such cells can be easily inserted into the system.

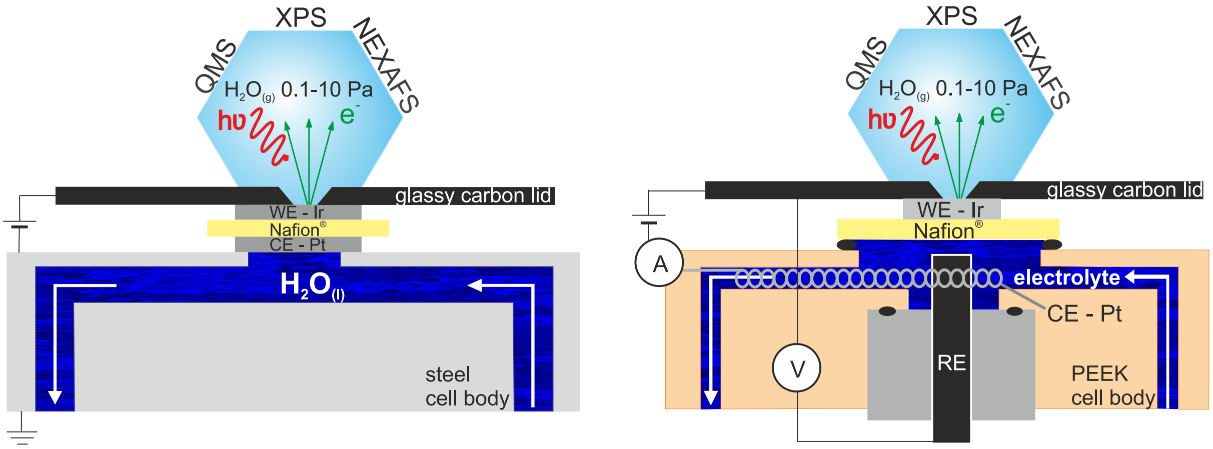

Figure S1: (left) Two-electrode *in situ* cell with sputtered Ir working and Pt counter electrode. (right) Three-electrode *in situ* cell with sputtered Ir working, Pt wire counter, and Ag/AgCl reference electrode. In both cells, water supplied from the rear diffuses through the desiccation cracks of the sputtered electrodes and the PEM and delivers the reactant molecules to the reaction chamber. While XPS and NEXAFS are measured, the gas composition is monitored by an on-line QMS. Through the connection to an external potentiostat, OER-relevant potentials can be applied to the working electrode.

In a first advancement, we upgraded the water supply from a batch reservoir to a continuous flow (see Figure S1 (left)). This continuous flow of water prevents the PEM from drying out and ensures a stable supply of reactant molecules to the working electrode throughout the experiments. This first upgrade permits measurement durations of several hours.

In a second advancement, we equipped the cell with a Ag/AgCl micro reference electrode (DRIREF-2SH, World Precision Instruments, USA) to work under well-defined potential conditions (see Figure S1 (right)). During this second upgrade, we needed to slightly modify the cell design: To allow the reference electrode to be located closer to the working electrode than the counter electrode, we replaced the sputtered Pt film by an externally inserted Pt wire. In addition, instead of water, the three-electrode cell requires

an electrolyte for proton conductivity. We used 0.1 M H_2SO_4 prepared from concentrated sulfuric acid (EMSURE®, 95-97 %, Merck KGaA, Darmstadt, Germany) and ultra-pure Milli-Q water (18.2 MΩ). For better corrosion stability, we replaced the stainless steel of the cell body by polyether ether ketone (PEEK).

For both cells, we realized the electrical contact to the working electrode (WE) via a glassy carbon lid. The use of this electrochemically resistant material prevents the strong corrosion of the lid material, which we had observed for a previously used stainless steel lid. In the two-electrode cell, we contacted and grounded the counter electrode (CE) via the stainless steel body while in the three-electrode cell, we contacted the CE directly with the Pt wire. The Ag/AgCl reference electrode (RE) was directly connected to the potentiostat.

As potentiostat, we used an SP-300 modular research grade device from Bio-Logic Science Instruments SAS, France. For the two-electrode cell, we operated the potentiostat in floating mode, since the CE was grounded via the stainless steel cell body in contact with the spectrometer. By this electrical connection between the CE and the spectrometer, their Fermi levels were aligned. Shifts observed in the BE of the recorded spectra could therefore be directly related to the potential difference between the WE and CE during the chronoamperometric (CA) measurements. For the three-electrode setup, we used the potentiostat in grounded mode.

For the samples, we used Nafion® 117 (AlfaAesar) as PEM throughout all of our experiments. The diameter of the circular samples was 12 mm. Prior to the deposition of the electrode materials, the Nafion® 117 was first purified in 3 vol.% H_2O_2 (prepared from 30 % H_2O_2 ROTIPURAN®, Carl Roth, Germany and Milli-Q water) for 2 h at 80 °C and then activated in 0.5 M H_2SO_4 (prepared from H_2SO_4 EMSURE®, 95-97 %, Merck KGaA, Darmstadt, Germany and ultra-pure Milli-Q water) for 2 h at 80 °C. Between and after these steps, the membranes were rinsed with Milli-Q water and finally dried and stored in air between clean filter paper.

We sputter-deposited the Ir and Pt films from metallic targets (Ir 99.99 % and Pt 99.99 %, Elektronen-Optik-Service GmbH, Germany) in 10 Pa Ar at 40 mA using a Cressington 208HR sputter coater. The deposition time was 180 s or 60 s for Ir and 120 s for Pt, resulting in film thicknesses ranging from 10 nm - 20 nm. The areas of the circular Ir and Pt electrodes were 6 mm and 9 mm, respectively. We used the working electrode size of 6 mm to determine the current densities from the measured currents. This determination is obviously just an approximation since we do not know the electrochemically active surface area from this electrode size.

We determined the morphologies of the sputtered films and their metallic distribution in a scanning electron microscope (SEM) Hitachi S-4800 FEG equipped with a Bruker XFlash detector and an energy dispersive X-ray spectroscopy (EDX) system Quantax.

The images were taken with an acceleration voltage of 1.5 kV in SE mode and the metallic distribution was determined via an X-ray map at 15 kV. We further investigated their nanostructure by TEM using an FEI TITAN 80-300 with an acceleration voltage of 200 kV. The SEM images in Figure S2 display the Ir and Pt films that have desiccation cracks, which enable the water transport across the electrode-membrane assembly. The X-ray scans in Figure S3 confirm the homogeneous distribution of the electrode materials on the Nafion® 117. The TEM images confirm the Ir film thickness of ≈20 nm and that the nanostructure of the film is composed of interconnected nanoparticles. These connected nanoparticles ensure the conductivity necessary for driving electrochemical experiments and measuring XPS.

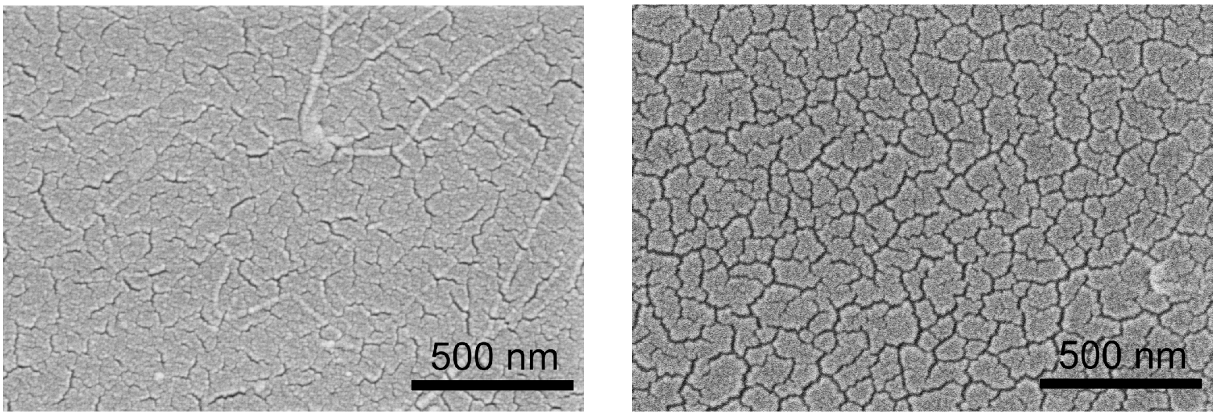

Figure S2: SEM images of (left) Ir and (right) Pt sputter-deposited on Nafion® 117. The images clearly show the desiccation cracks of the sputter-deposited films allowing for an efficient water transport through the metallic films.

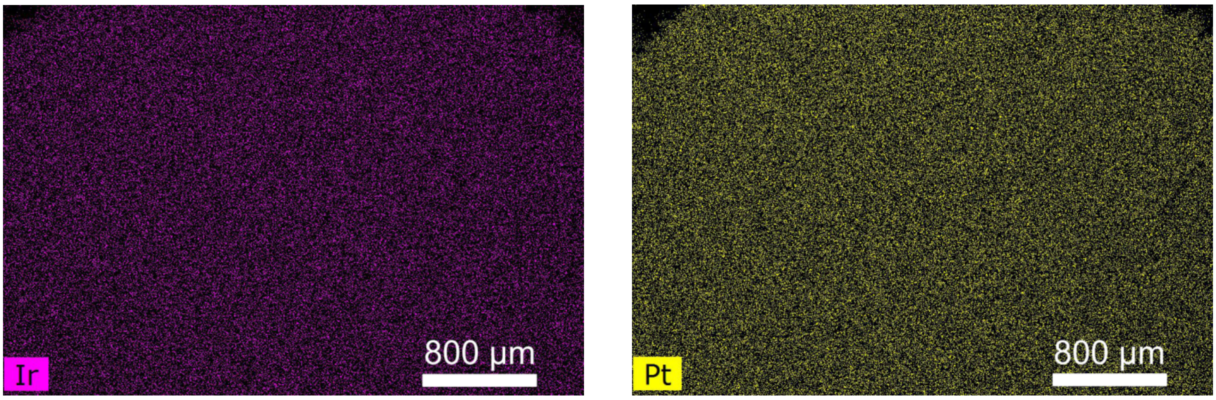

Figure S3: X-ray map of (left) Ir and (right) Pt sputter-deposited on Nafion® 117. The images show the homogeneous distribution of the electro-active materials.

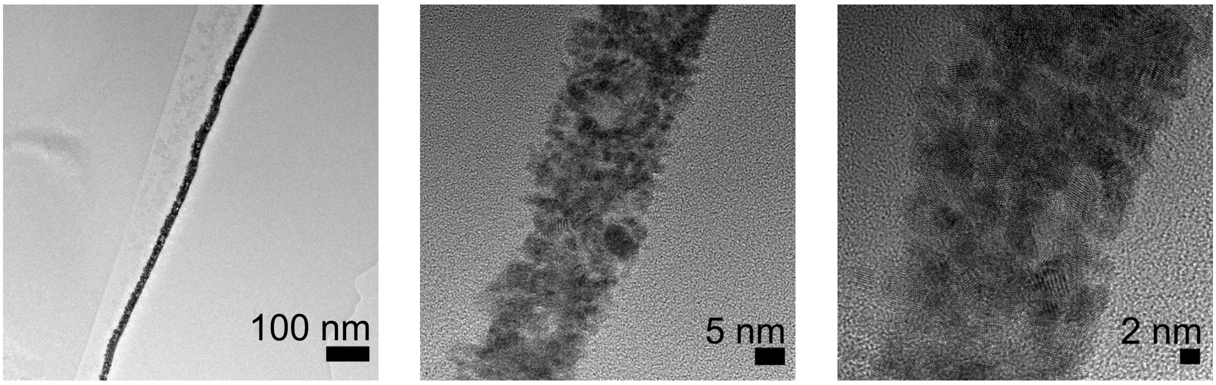

Figure S4: TEM cross sections of the sputter-deposited Ir in different magnifications.

Figure S5 shows an XPS survey of such a sputter-deposited Ir film on Nafion® 117. Apart from the iridium core levels, we also detect fluorine, oxygen, carbon and sulfur signals. These signals mainly originate from Nafion® 117 and the fluid electrolyte H_2SO_4. The reason for Nafion® 117 to contribute to the XPS signal is the mud-crack type structure of the sputter-deposited Ir film. In the desiccation cracks, the membrane is directly exposed to the X-rays and its emitted photoelectrons contribute to the overall signal, hence we are partly probing the triple phase boundary (electrolyte, water, iridium) of interest. Nevertheless, parts of the oxygen and carbon signal will also originate from surface oxidation of the Ir nanoparticles and carbonaceous contamination on the Ir surface. Since we do not observe any (differential) charging of the surface, we can be sure that the Ir islands are interconnected and form a conductive film.

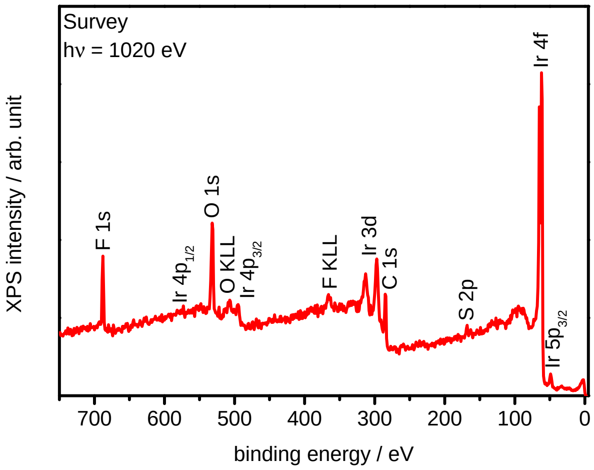

Figure S5: XPS survey of Ir-coated Nafion® 117 (60 s Ir sputtered, sample 23898) with an identification of the observed core levels. Recorded in the three-electrode cell at E_{oc} (ring current=13 mA, p=0.45 Pa, 0.1 M H_2SO_4).

3 Control investigation of uncoated PEM

To ensure that the registered signals and main spectral regions of interest were neither distorted nor affected by signals of the substrate Nafion® 117 membrane, uncoated membranes were investigated in control experiments.
For the first investigation, a purified and activated plain Nafion® 117 was mounted in the three-electrode cell. In a first step, a cyclic voltammogram (CV) was recorded (see Figure S6 (left)). The CV shows reversible oxidation/reduction waves at 0.6 V vs. SHE and 0.5 V vs. SHE, respectively. In the OER-relevant region of the CV starting from 1.5 V vs. SHE only a slight current increase is observed. For comparison, the CV of an Ir-coated sample is shown in Figure S6 (right). Comparing these CVs, on the one hand, we see that the reversible oxidation/reduction signals of Nafion® 117 are still slightly visible for Ir-coated sample. On the other hand, we see that, in addition, the characteristic oxidation signals of iridium at ≈1 V vs. SHE and ≈1.4 V vs. SHE are present and that both the capacitative currents and the current increase in the OER region are almost one order of magnitude higher when Ir is present.

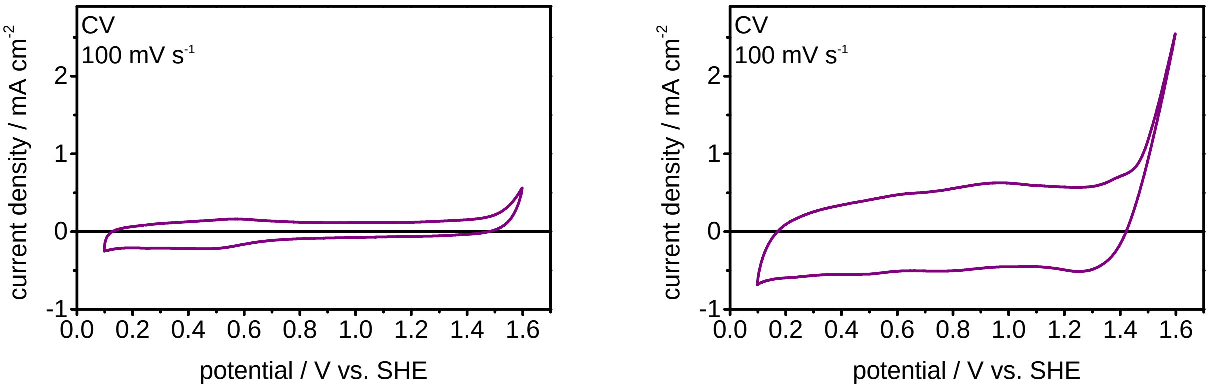

Figure S6: Cyclic voltammograms of (left) uncoated Nafion® 117 (sample 23879, p=16.5 Pa) and (right) Ir-coated Nafion® 117 (180 s Ir sputtered, sample 23878, p=5.8 Pa) recorded in the three-electrode cell prior to chronoamperometry (scan rate=100 mV s^{-1}, 0.1 M H_2SO_4).

A similar observation holds for the comparison of the current density and oxygen QMS traces measured during different applied potentials to uncoated and Ir-coated Nafion® 117 (see Figure S7): The current density measured for the uncoated is considerably lower than for the Ir-coated Nafion® 117. The oxygen QMS trace of plain Nafion® 117 is not perturbed when the potential is raised above OER-relevant values, i. e. no oxygen evolves from the uncoated Nafion® 117, while the oxygen QMS signal considerably increases for the Ir-coated Nafion® 117 with each potential increase.

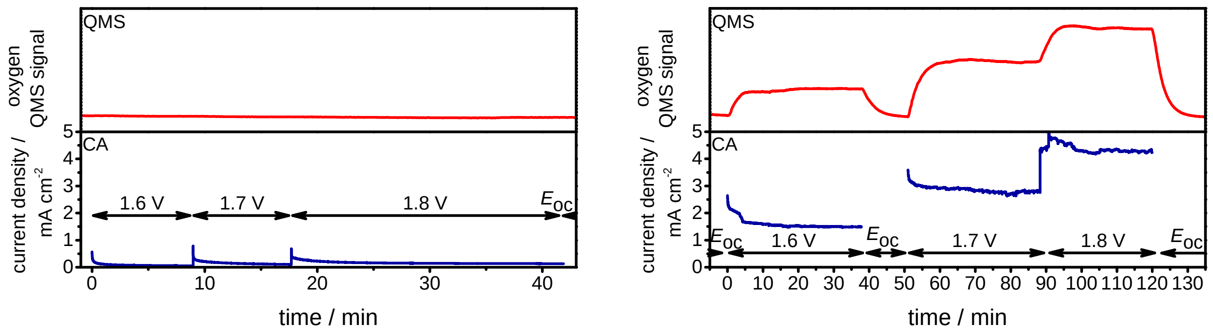

Figure S7: (bottom) Chronoamperometry and (top) oxygen QMS signal of (left) uncoated Nafion® 117 (sample 23879, p=16.5 Pa) and (right) Ir-coated Nafion® 117 (180 s Ir sputtered, sample 23878, p=5.8 Pa) recorded in the three-electrode cell with the indicated potentials vs. SHE applied (0.1 M H_2SO_4).

Finally, the O K-edge of plain Nafion® 117 was measured at the different applied potentials. Figure S8 shows the collected AEY (left) and TEY (right) data. In the AEY, the signal at ≈532 eV may result from carbonaceous contamination while the large signal at ≈537 eV originates from sulfates from the Nafion® 117 and the used electrolyte H_2SO_4. In the TEY, the most prominent signals descend from the gas-phase resonances of water vapor.[7] From both graphs, we observe that our main region of interest, the excitation energy values of 529 eV and 530 eV, seems to be unaffected by the background signals. A zoom into this region confirms the absence of disturbing background signals (see Figure S9).

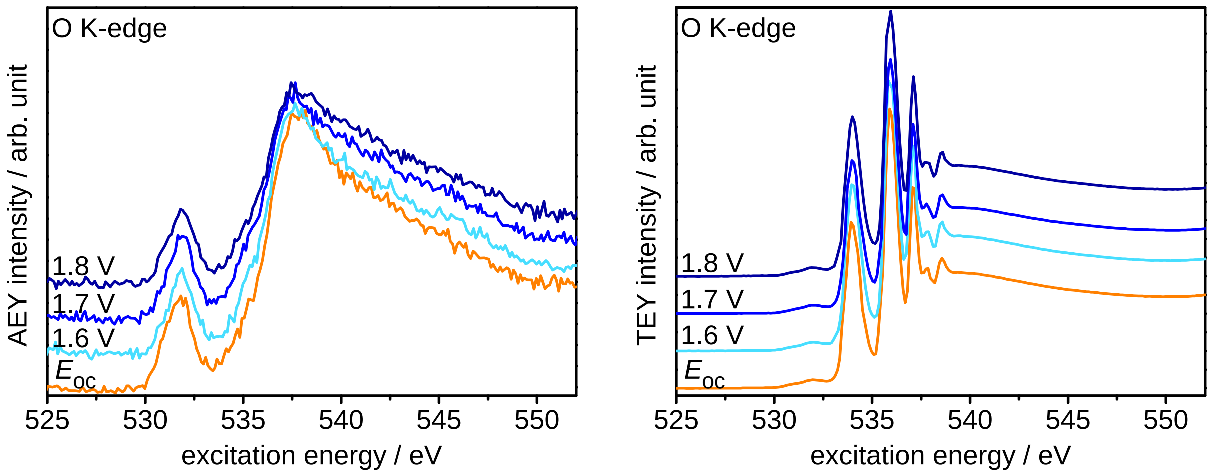

Figure S8: (left) AEY and (right) TEY signals of the O K-edges of uncoated Nafion® 117 (sample 23879), consecutively recorded (bottom to top) in the three-electrode cell with the indicated potentials vs. SHE applied (ring current=70 mA, p=16.5 Pa, 0.1 M H_2SO_4).

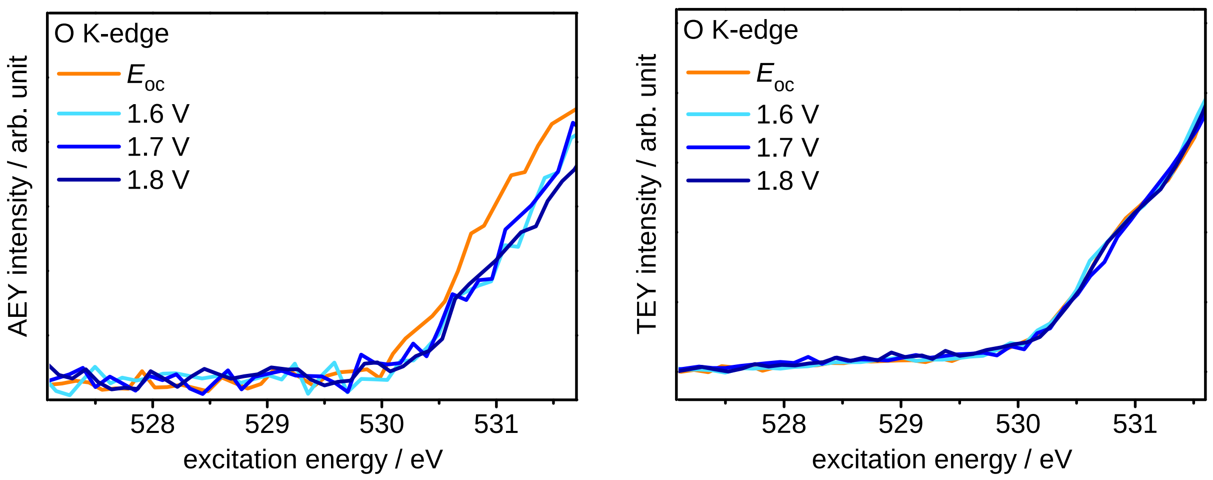

Figure S9: Zoomed low excitation energy regions of the (left) AEY and (right) TEY signals of the O K-edges of uncoated Nafion® 117 (sample 23879), consecutively recorded (E_{oc} to 1.8 V) in the three-electrode cell with the indicated potentials vs. SHE applied (ring current=70 mA, p=16.5 Pa, 0.1 M H_2SO_4).

Although the water supply from the back of the membrane is continuous, we always observe slight alterations of the pressure in the measurement compartment and therefore also in the amount of water present in the gas phase. Especially when comparing a sequence of measurements, the tails of the water signals may affect the background and distort the iridium oxide signals. Hence, the water vapor background signals observed in the TEY measurement may still become disturbing to the interpretation of the spectra, even though Nafion® 117 and water do not have disturbing signals exactly at the excitation energies of interest. To minimize the influence of differing background signals on the spectra, we reduced the pressure in the measurement compartment by increasing the applied pumping speed of the NAP-XPS system. At a pressure of ≈0.1 Pa, the contribution of the water gas phase is no longer visible in the TEY measurement of pure Nafion® 117 (see Figure S10) and therefore the signal background is no longer dependent on the gas-phase water pressure and less sulfate is expected to deposit on the surface.

The advantage of using the TEY instead of the AEY signal is the increased signal-to-noise ratio of the TEY measurements. Therefore, for the controlled measurements collected near the onset of iridium's OER activity with low ring currents to prevent beam damage, it is necessary to rely on the TEY measurements. As will be shown later, we still observe the evolution of oxygen at these reduced pressure conditions by means of QMS, hence the device is still working under relevant conditions at these reduced pressures.

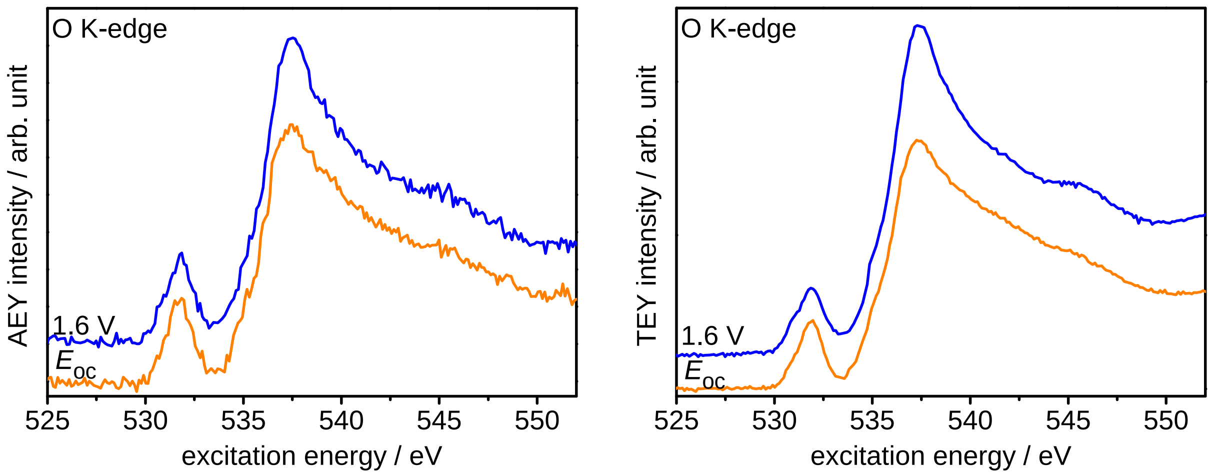

Figure S10: (left) AEY and (right) TEY signals of the O K-edges of uncoated Nafion® 117 (sample 23896), consecutively recorded (bottom to top) in the three-electrode cell with the indicated potentials vs. SHE applied (ring current=50 mA, p=0.1 Pa, 0.1 M H_2SO_4).

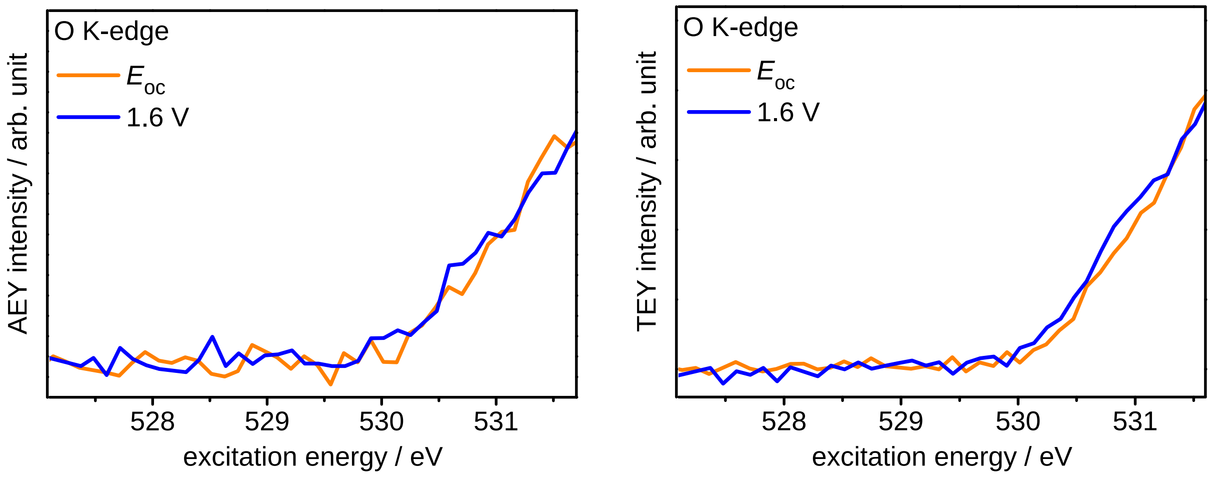

Figure S11: Zoomed low excitation energy regions of the (left) AEY and (right) TEY signals of the O K-edges of uncoated Nafion® 117 (sample 23896), consecutively recorded (E_{oc} to 1.6 V) in the three-electrode cell with the indicated potentials vs. SHE applied (ring current=50 mA, p=0.1 Pa, 0.1 M H_2SO_4).

4 Ir 4f fit parameters

The fitting of the Ir 4f spectra shown in Figure 1 was done using the fit model previously derived for iridium and its oxides.[8,9] The parameters employed for the fits shown in Figure 1 are given in Table S1.

Table S1: Fit parameters of Ir 4f spectra recorded *in situ* at the indicated potentials with a kinetic energy of the photoelectrons of 450 eV. FWHM, full width at half maximum; BE, binding energy; E_{oc}, open circuit potential; DS, Doniach-Šunjić; GL, Gauss-Lorentz; SGL, Gaussian-Lorentzian sum form.

	Ir 4f$_{7/2}$ Ir^0	Ir 4f$_{5/2}$ Ir^0	Ir 4f$_{7/2}$ Ir^{IV}	Ir 4f$_{5/2}$ Ir^{IV}	Ir 4f$_{7/2}$ Ir^{IV} sat1	Ir 4f$_{5/2}$ Ir^{IV} sat1	Ir 4f$_{5/2}$ Ir^{IV} sat2	Ir 4f$_{7/2}$ Ir^{III}	Ir 4f$_{5/2}$ Ir^{III}	Ir 4f$_{7/2}$ Ir^{III} sat1	Ir 4f$_{5/2}$ Ir^{III} sat1
E_{oc}											
line	DS(0.132,163)	DS(0.132,163)	DS(0.2,100)	DS(0.2,100)	GL(0)	GL(0)					
shape	SGL(100)	SGL(100)	SGL(45)	SGL(45)							
area/%	54.0	38.0	3.8	2.9	0.7	0.6					
FWHM/eV	0.8	0.7	0.9	1.0	3.1	3.1					
BE/eV	60.8	63.8	61.8	64.8	62.8	65.8					
2 V											
line	DS(0.132,163)	DS(0.132,163)	DS(0.2,100)	DS(0.2,100)	GL(0)	GL(0)					
shape	SGL(100)	SGL(100)	SGL(45)	SGL(45)							
area / %	45.9	32.3	10.4	7.9	2.0	1.5					
FWHM/eV	0.8	0.8	0.9	1.0	3.1	3.1					
BE/eV	60.8	63.8	61.8	64.8	62.8	65.8					
2.5 V											
line	DS(0.132,163)	DS(0.132,163)	DS(0.2,100)	DS(0.2,100)	GL(0)	GL(0)	GL(0)	DS(0.2,100)	DS(0.2,100)	GL(0)	GL(0)
shape	SGL(100)	SGL(100)	SGL(45)	SGL(45)				SGL(45)	SGL(45)		
area/%	7.2	5.1	36.1	27.2	7.0	5.3	0.2	5.8	4.7	0.8	0.6
FWHM/eV	0.7	0.8	1.1	1.1	2.9	2.9	2.5	0.7	0.8	2.9	2.9
BE/eV	60.8	63.8	61.8	64.8	62.8	65.8	67.8	62.3	65.3	63.3	66.3

5 *In situ* investigation near the onset of iridium's OER activity

The controlled measurements near the onset of iridium's OER activity were all performed with the three-electrode cell at reduced pressures (≈0.1 Pa). To minimize the beam damage of the beam sensitive 529 eV feature of the O^{I-} species, we performed these measurements in the low-alpha mode of BESSY II with reduced ring currents. The pressure and exact ring current conditions will always be denoted in the figure captions.

To precondition and activate the Ir films on Nafion® 117, we always performed a sequence of 35 CVs between 0.1 V vs. SHE and 1.6 V vs. SHE prior to all other measurements. Subsequently, we recorded a scan of the O K-edge, an XPS survey and the Ir 4f, C 1s, and O 1s core levels at E_{oc} to capture the initial state of the Ir electrode surface. Finally, we applied OER-relevant potentials, monitored the corresponding current densities of the WE and oxygen QMS traces and recorded NEXAFS and XPS to observe changes in the electronic structure of the iridium electrodes. In the following, we will show three examples of typical experiment results and how the oxygen evolution rate and current density are related with the presence of O^{I-} species on the Ir electrode surface.

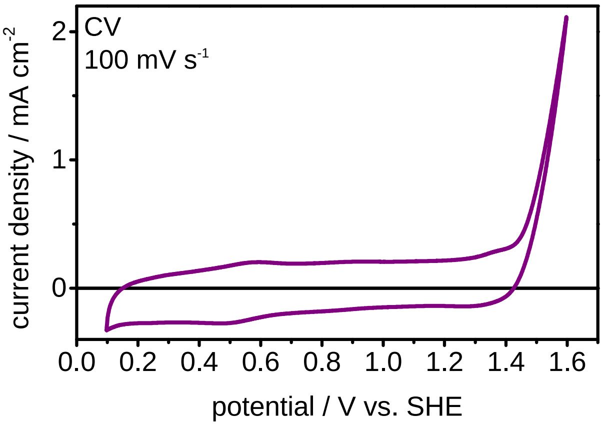

Figure S12: Cyclic voltammogram of Ir-coated Nafion® 117 (180 s Ir sputtered, sample 23894) recorded in the three-electrode cell with the indicated potentials vs. SHE applied (p=0.5 Pa, 0.1 M H_2SO_4).

Figures S12 and S13 show the CV and the subsequently recorded CA of sample 23894, a Nafion® 117 that was sputter-coated for 180 s with metallic Ir. The CV mainly shows the oxidation/reduction signal of Nafion® 117 at ≈0.6 V vs. SHE, a slight indication of the Ir-oxidation signals at 1 V vs. SHE and 1.4 V vs. SHE and the OER onset at around 1.5 V vs. SHE. When OER-relevant potentials are applied, the current density in the CA increases stepwise with each potential increase. A concomitant stepwise increase is

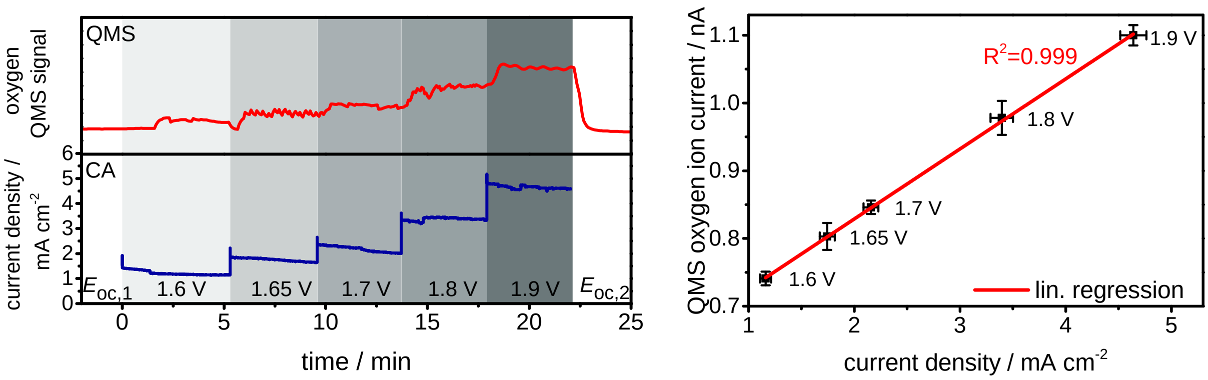

Figure S13: (left) Chronoamperometry (bottom) and oxygen QMS signal (top) and (right) linear correlation between current density and evolved oxygen of Ir-coated Nafion® 117 (180 s Ir sputtered, sample 23894) recorded in the three-electrode cell with the indicated potentials vs. SHE applied (p=0.5 Pa, 0.1 M H_2SO_4).

observed in the oxygen evolution rate as mirrored in the the oxygen QMS signal. We observe a linear relation between the current density and the oxygen evolution activity. When the potential is turned off, the oxygen signal immediately drops back to its original value.

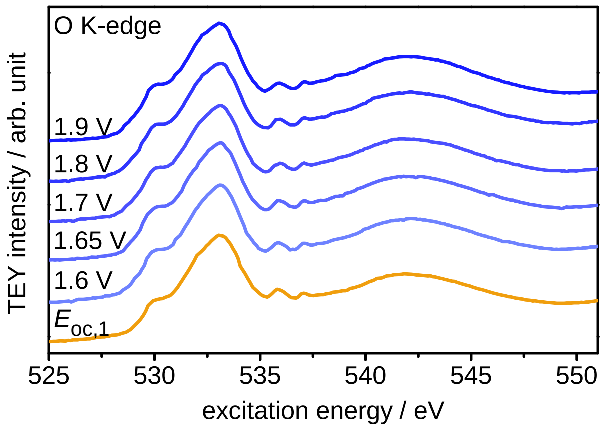

Figure S14: O K-edges of Ir-coated Nafion® 117 (180 s Ir sputtered, sample 23894), consecutively recorded (bottom to top) in the three-electrode cell with the indicated potentials vs. SHE applied (ring current=70 mA, p=0.5 Pa, 0.1 M H_2SO_4).

Figure S14 displays the O K-edges recorded at consecutively applied potentials. At the pressure established during this experiment, the O K-edge still shows minor resonances of gas-phase water. Nevertheless, these resonances do not severely influence the spectra. In this representation, we observe nearly no changes in the spectra in dependence of the applied potential. However, when we zoom into the region of interest at low excitation energies, we do observe clear changes depending on the applied potential (see Figure S15). To quantify the observed changes, we used the spectra calculated[8] for O^{I-} and O^{II-} (shown

in Figure S27) to fit the low excitation energy region of the O K-edge. We obtain good agreement between the measured spectra and the resulting fit envelope (see Figure S15).

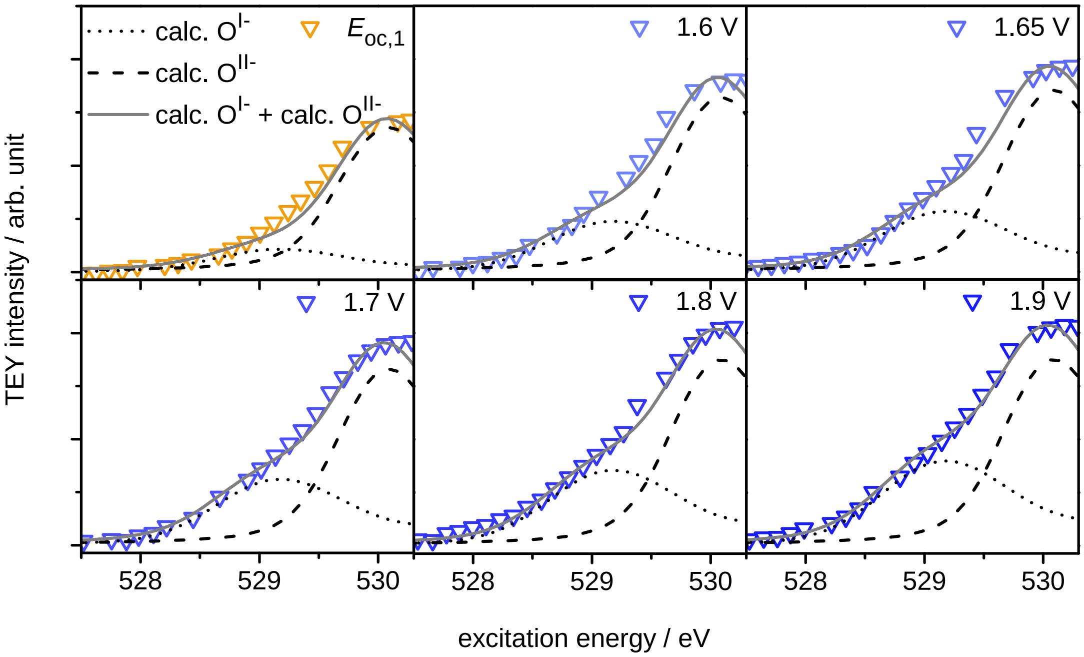

Figure S15: Zoomed and fitted low excitation energy regions of O K-edges of Ir-coated Nafion® 117 (180 s Ir sputtered, sample 23894) consecutively recorded (left to right, top to bottom) in the three-electrode cell with the indicated potentials vs. SHE applied (ring current=70 mA, p=0.5 Pa, 0.1 M H_2SO_4).

In a next step, we wanted to identify the relation between the observed oxygen species and the oxygen evolution activity of the electrode. For this purpose, we plotted the relative concentration of O^{I-} and O^{II-} against the current density recorded with the potentiostat and the oxygen ion current registered by QMS, respectively (see Figure S16). The determined error values originate from the fluctuations in measured current densities (x-error) and the uncertainties in peak height determination (y-error). We observe a linear relationship between the O^{I-}-species and both the current density measured with the potentiostat and the oxygen ion current registered by QMS (R^2-values of 0.94 and 0.95). In contrast, we observe only a loose dependence of the O^{II-} concentration on current density and ion current (R^2-values of 0.66 and 0.67).

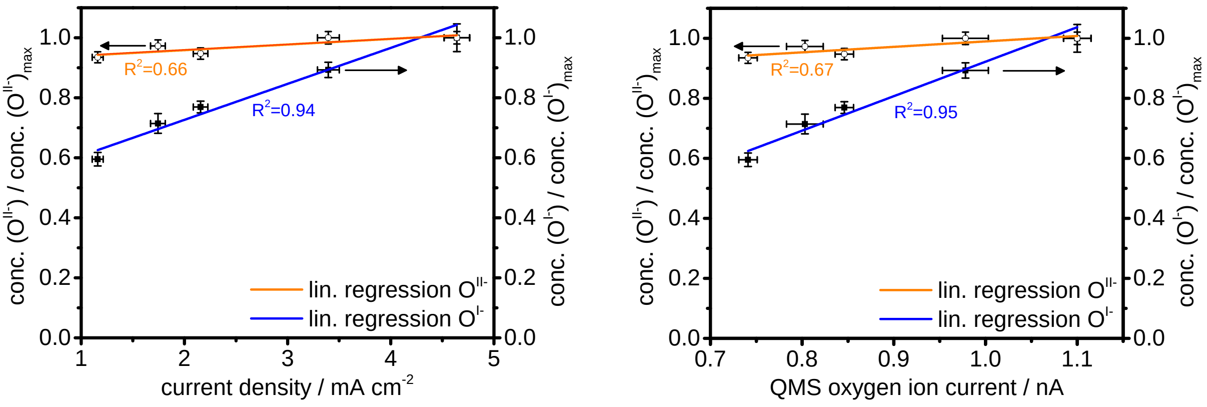

Figure S16: Normalized O^{I-} and O^{II-} concentrations over (left) current density and (right) QMS oxygen ion current of Ir-coated Nafion® 117 (180 s Ir sputtered, sample 23894) at consecutively applied potentials between 1.6 V vs. SHE and 1.9 V vs. SHE.

To confirm the results obtained with sample 23894, we repeated the experiments with sample 23895, which was also a 180 s Ir-sputtered Nafion® 117 from the same batch of sample. Figures S17 to S21 show the same features and trends as observed in the previous experiment: The CV in Figure S17 counts with the oxidation waves of iridium oxides at 1 V vs. SHE and 1.4 V vs. SHE and the additional reversible oxidation/reduction feature of Nafion® 117 at ≈0.6 V vs. SHE. The CA and the QMS oxygen ion current in Figure S18 show a linear increase with increasing potential applied to the Ir WE. While the overview spectrum of the O K-edge in Figure S19 does not show marked changes during the experiment, a zoom in the low excitation energy region and the corresponding fits in Figure S20 shows a clear increase of O^{I-} concentration with increasing potential.

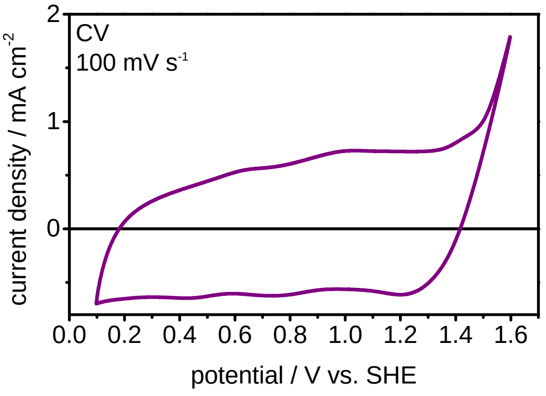

Figure S17: Cyclic voltammogram of Ir-coated Nafion® 117 (180 s Ir sputtered, sample 23895) recorded in the three-electrode cell with the indicated potentials vs. SHE applied (p=0.3 Pa, 0.1 M H_2SO_4).

A quantification of the relative O^{I-} and O^{II-} concentrations and their plots against the current density measured with the potentiostat and the oxygen ion current determined

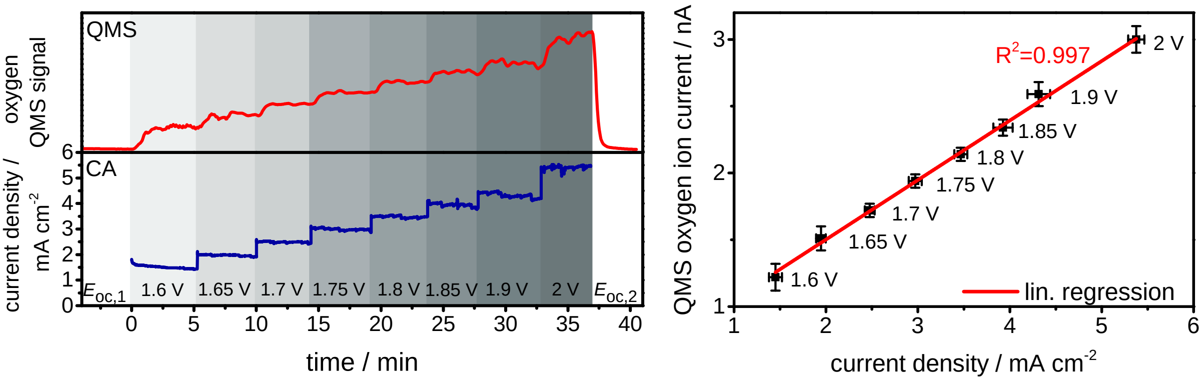

Figure S18: (left) Chronoamperometry (bottom) and oxygen QMS signal (top) and (right) linear correlation between current density and evolved oxygen of Ir-coated Nafion® 117 (180 s Ir sputtered, sample 23895) recorded in the three-electrode cell with the indicated potentials vs. SHE applied (p=0.3 Pa, 0.1 M H_2SO_4).

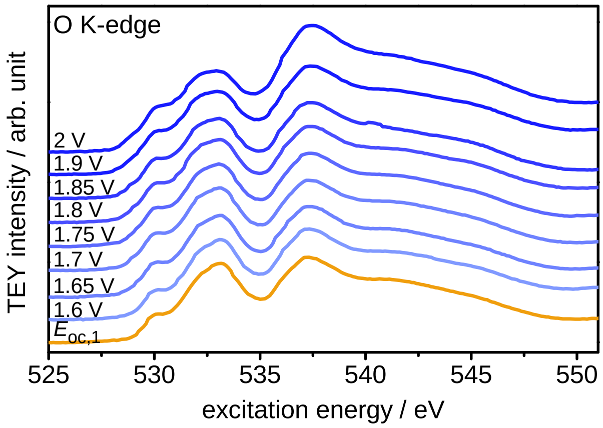

Figure S19: O K-edges of Ir-coated Nafion® 117 (180 s Ir sputtered, sample 23895), consecutively recorded (bottom to top) in the three-electrode cell with the indicated potentials vs. SHE applied (ring current=60 mA, p=0.3 Pa, 0.1 M H_2SO_4).

by QMS in Figure S21 confirm the linear dependence of oxygen evolution activity and O^{I-} concentration.

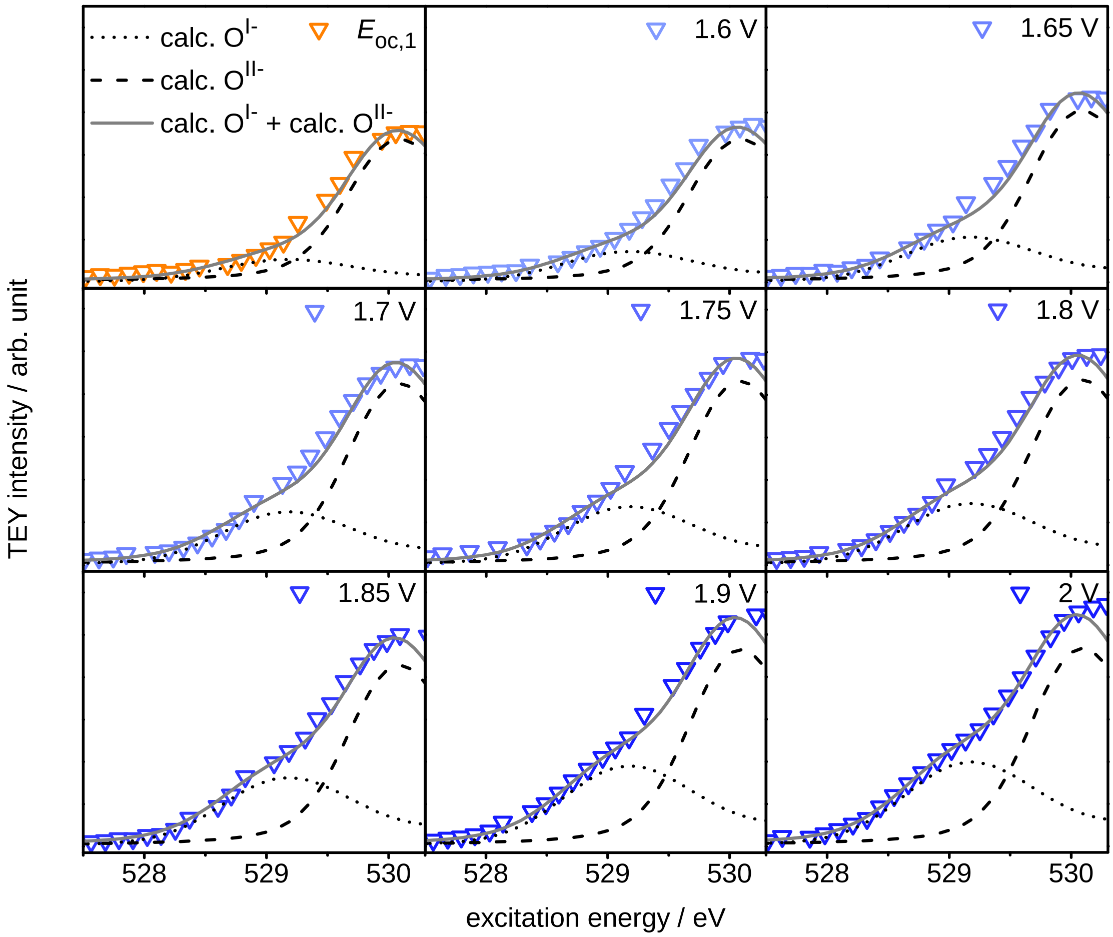

Figure S20: Zoomed and fitted low excitation energy regions of O K-edges of Ir-coated Nafion® 117 (180 s Ir sputtered, sample 23895), consecutively recorded (left to right, top to bottom) in the three-electrode cell with the indicated potentials vs. SHE applied (ring current=60 mA, p=0.3 Pa, 0.1 M H_2SO_4).

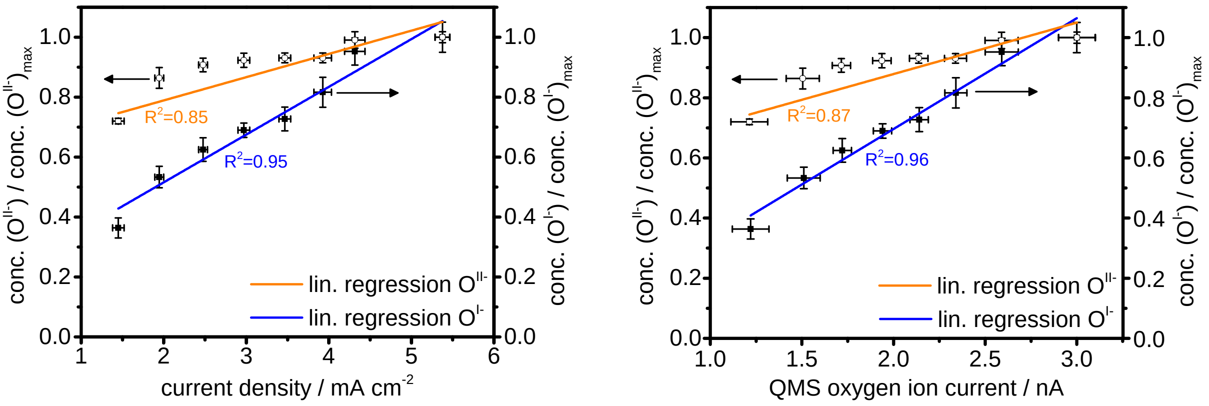

Figure S21: Normalized O^{I-} and O^{II-} concentrations over (left) current density and (right) QMS oxygen ion current of Ir-coated Nafion® 117 (180 s Ir sputtered, sample 23895) at consecutively applied potentials between 1.6 V vs. SHE and 2 V vs. SHE.

In a final *in situ* investigation, we tested the stability of the O^{I-} species and alternatively switched on and off the potential applied to the Ir WE. We first confirmed the similar behavior of the Ir-coated Nafion® 117 (sample 23898, 60 s Ir sputtered) in cyclic voltammetry and obtained a similar CV shape as for the previous samples (see Figure S22 (left)).

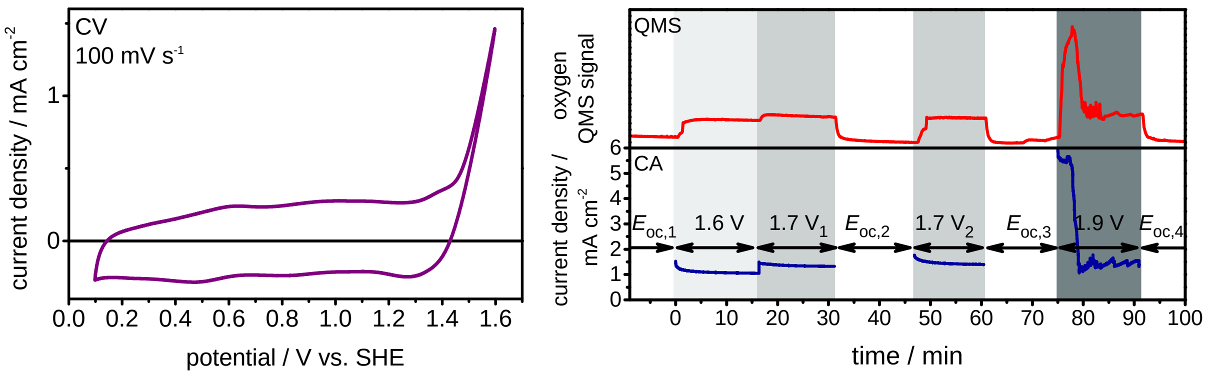

Figure S22: (left) Cyclic voltammogram and (right) chronoamperometry (bottom) and oxygen QMS signal (top) of Ir-coated Nafion® 117 (60 s Ir sputtered, sample 23898) recorded in the three-electrode cell with the indicated potentials vs. SHE applied (p=0.45 Pa, 0.1 M H_2SO_4).

We then applied OER-relevant potentials of 1.6 V vs. SHE, 1.7 V vs. SHE, and 1.9 V vs. SHE and turned off the potential in between. The resulting current densities and QMS oxygen ion currents are shown in Figure S22 (right). At 1.9 V vs. SHE the current density and the corresponding oxygen ion current increase sharply for a short period of time, in which the electrode possibly reaches a highly active state. Due to the short time period, it was not possible to record the corresponding O K-edge. The measurement at 1.9 V vs. SHE was recorded from 80 min onwards.

In the overview spectra of the O K-edge, we can observe already in this representation that the 529 eV species is switched on and off with the applied potential (see Figure S23). This observation becomes even clearer when considering the zoomed in and fitted low excitation energy region in Figure S24.

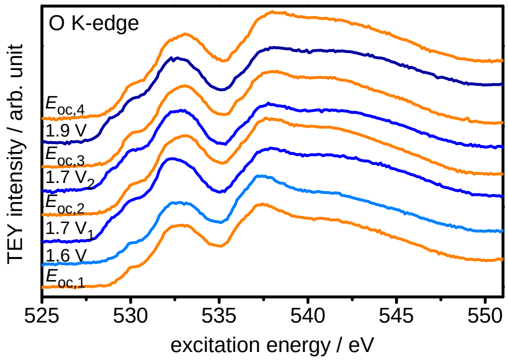

Figure S23: O K-edges of Ir-coated Nafion® 117 (60 s Ir sputtered, sample 23898), consecutively recorded (bottom to top) in the three-electrode cell with the indicated potentials vs. SHE applied (ring current=13 mA, p=0.45 Pa, 0.1 M H_2SO_4).

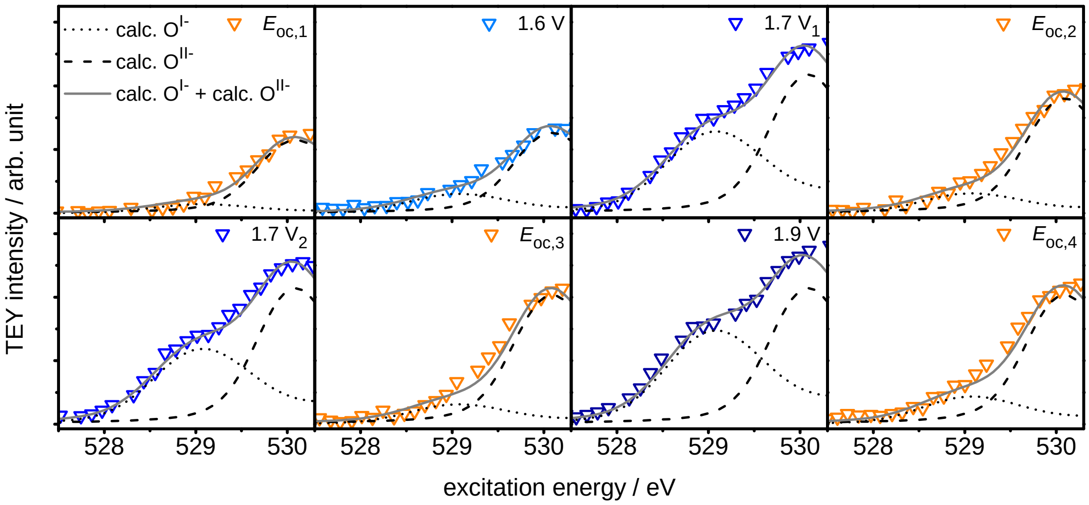

Figure S24: Zoomed and fitted low excitation energy regions of O K-edges of Ir-coated Nafion® 117 (60 s Ir sputtered, sample 23898), consecutively recorded (left to right, top to bottom) in the three-electrode cell with the indicated potentials vs. SHE applied (ring current=13 mA, p=0.45 Pa, 0.1 M H_2SO_4).

Figures S25 and S26 show the corresponding Ir 4f and O 1s spectra of sample 23898 recorded at the different applied potentials after the initial pre-treatment of 35 CVs. Although the Ir films were subject to potential cycling prior to the first measurement at E_{oc}, expected to lead to the formation of an oxidic overlayer, the spectrum is dominated by metallic Ir and only little intensity is observed at higher binding energy alluding to oxidized iridium. This observation can be understood since only a few layers of oxidized material cover the metallic support. The thickness of this oxide layer is significantly smaller than the probing depth of our Ir 4f XPS measurements of approximately 2 nm. Therefore, the spectra recorded in the three-electrode cell at E_{oc} are still be dominated by the metallic Ir signal of the metallic "support". The major change observed in the Ir 4f spectrum occurs at the first application of an OER-relevant potential of 1.7 V vs. SHE. We observe slightly more intensity at higher binding energy, suggesting a slight surface

oxidation, which is in line with the observation of the increasing contribution of O^{I-} and O^{II-} at this applied potential (see Figure S24). Subsequent potential cycles of turning the applied potential on and off have nearly no impact on the shape of the Ir 4f spectrum. In the O 1s spectra, complementary to the O K-edge, we observe an increased intensity at lower binding energies of 529 eV, where the O^{I-} are located, while the OER proceeds. Since this sample was measured at a low storage ring current of 13 mA, the signal-to-noise ration of these spectra is rather poor and we concentrated our interpretation on the O K-edge. Nevertheless, the O 1s spectra confirm the trends observed in the O K-edge.

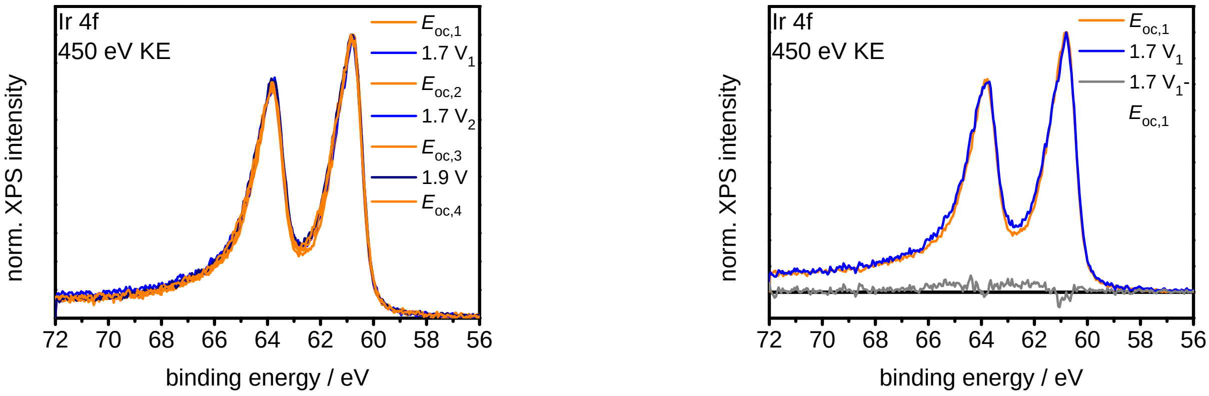

Figure S25: Ir 4f signals of Ir-coated Nafion® 117 (60 s Ir sputtered, sample 23898) recorded in the three-electrode cell with the indicated potentials vs. SHE applied (p=0.45 Pa, 0.1 M H_2SO_4).

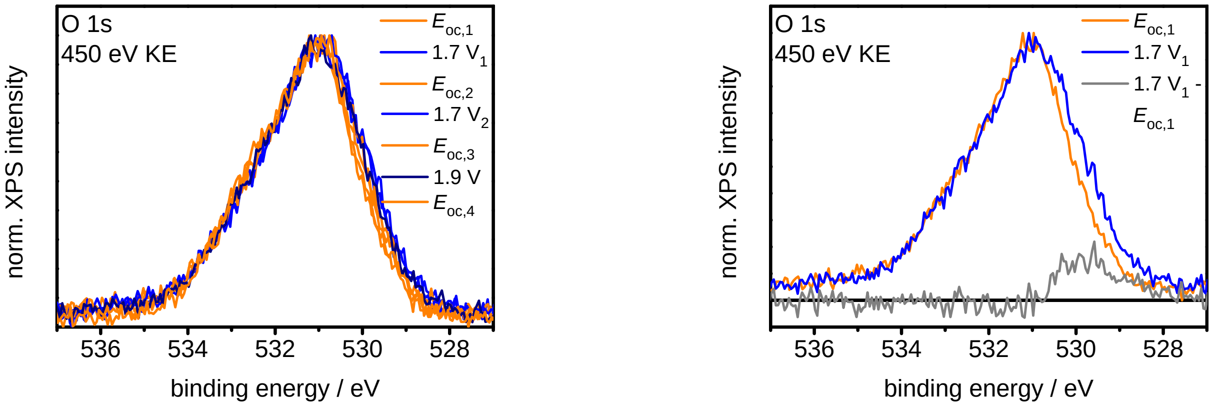

Figure S26: O 1s signals of Ir-coated Nafion® 117 (60 s Ir sputtered, sample 23898) recorded in the three-electrode cell with the indicated potentials vs. SHE applied (p=0.45 Pa, 0.1 M H_2SO_4).

6 Calculation details

As described in detail in our previous work,[8,9] density functional theory (DFT) calculations were performed using the Quantum ESPRESSO package version 5.3.0[10] with the Perdew, Burke, and Ernzerhof (PBE) exchange and correlation potential.[11] Ultrasoft pseudopotentials were taken from the PSlibrary for all total energy calculations.[12] A kinetic energy cutoff of 30 Ry and a charge density cutoff of 300 Ry was used in all calculations. A **k**-point mesh equivalent to (8x4x1) was employed for the surface unit cells. Surfaces were modeled using 5 layers of the crystallographic unit. Oxygen K-edge spectra were computed using a one-electron Fermi's golden rule expression as implemented in the XSpectra package.[13] A Lorentzian with an energy dependent linewidth, $\Gamma(E) = \Gamma_0 + \Gamma(E)$, was employed to account for lifetime broadening. The ΔSCF (self-consistent field) method was used to compute O 1s binding energies.

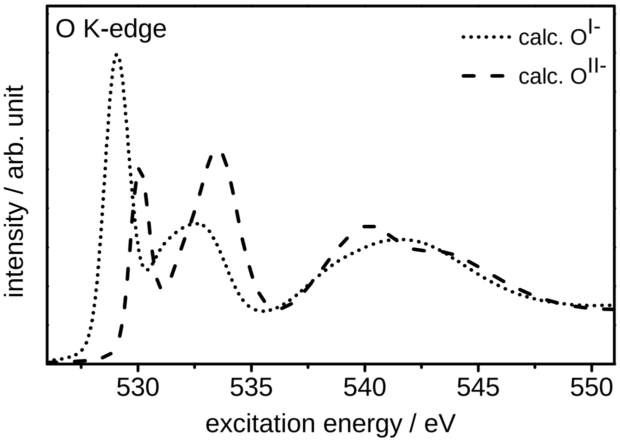

Figure S27: Calculated O K-edges of bulk O^{I-} and O^{II-} species.[8,9]

Figure S27 shows the calculated O K-edges of bulk O^{I-} and O^{II-} spectra[8,9] used to fit the low excitation energy region of the *in situ* measurements.

The potential at which O^{I-} forms on an iridium surface was computed using the DFT energies along with the well-known concept of a theoretical standard hydrogen electrode (SHE).[14] In this approach, we assume the surface is in thermodynamic equilibrium with protons and liquid water at 298 K at a fixed applied potential and pH. Thus, the surface can exchange oxygen and hydroxyl with the water. The potential and pH dependence of the free energy can be captured through the chemical potential of the proton and electron by writing:

$$2H^+_{(aq)} + 2e^- \leftrightarrow H_{2(g)}, \quad (1)$$

with $\Delta G^0 = 0$ at pH=0 and 10^5 Pa H_2 pressure. This allows us to set U=0 V vs. SHE. With this definition it is now straightforward to compute changes in Gibbs Free Energies as:

$$\Delta G = \Delta G_0 + \Delta G_U + \Delta G_{pH} \tag{2}$$

where ΔG_0, ΔG_U, and ΔG_{pH} take their standard definitions:

$$\Delta G_0 = \Delta E + \Delta ZPE - T\Delta S, \tag{3}$$

$$\Delta G_U = -eU, \tag{4}$$

and

$$\Delta G_{pH} = -k_B T \ln 10 pH. \tag{5}$$

Here ΔE is the reaction energy computed from DFT, ΔZPE and ΔS are the changes in zero point energy and entropy due to reaction, respectively. For the zero point energy (ZPE) term the vibrational modes of the solid are computed using DFT while the molecular ZPE and S are taken from tabulated data.

We investigated O- and OH-groups on the (110) and (100) surfaces of rutile-type IrO_2. Here we found that the μ_2-OH bridges are predicted to be deprotonated at $\approx$ 1.3 V vs. SHE on the (110) surface when μ_1-O is also present and $\approx$ 1.2 V vs. SHE when a μ_1-OH is present. In both cases μ_2-O is predicted to form, see O K-edges below. Similarly, at an applied potential of $\approx$ 1.2 V vs. SHE the μ_2-OH bridges on the (100) surface are predicted to transform into μ_2-O when a μ_1-OH is present. Deprotonation of the μ_1-OH is predicted to occur at $\approx$ 1.8 V vs. SHE on the (110) surface and $\approx$ 1.6 V vs. SHE on the (100) surface.

The simulated O K-edge spectra corresponding to the aforementioned structures suggest that the μ_2-O is seen during our experiments, which gives rise to a resonance at $\approx$529 eV, see Figures S28 and S29. The exact position of the resonance depends on surface termination and the nature of the coadsorbed species, with the resonance shifting to lower excitation energies when μ_1-OH or μ_1-O are also present. While we cannot rule out the presence of μ_1-OH (giving a resonance at $\approx$ 530 eV), we do not see any resonance at 528 eV in the experiments that would be indicative of μ_1-O.

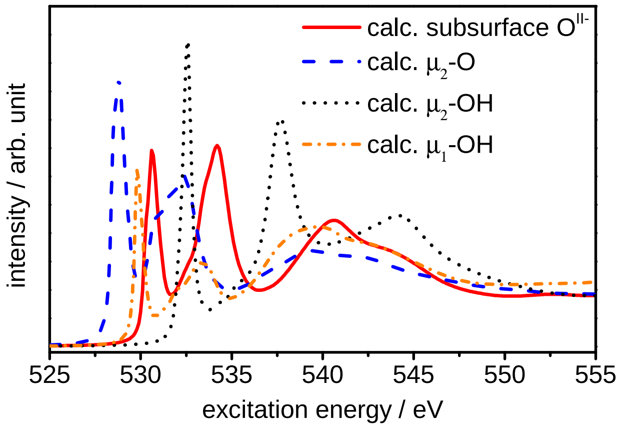

Figure S28: O K-edges computed for a subsurface O^{II-} in the third layer beneath the (110) surface (solid red) and a μ_2-O (dashed blue), a μ_2-OH (black dotted), and a μ_1-OH (dotted-dashed orange) on the (110) surface.

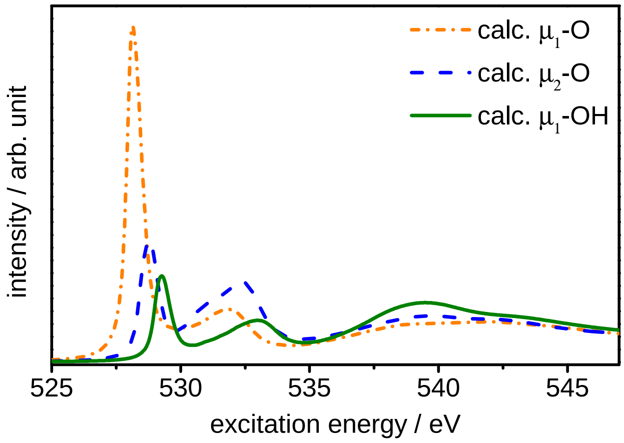

Figure S29: O K-edges computed for a μ_1-OH (dotted-dashed orange), a μ_2-O (dashed blue) and a μ_1-O (solid green) species on the (100) surface of rutile-type IrO_2.

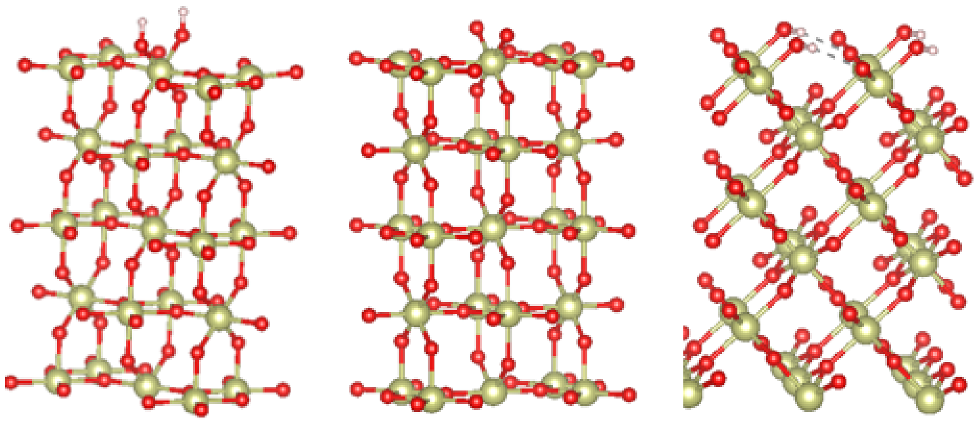

Figure S30: Ball and stick models of (110) surface with μ_2-OH (left) and μ_2-O (center) along with a model of a (100) surface with μ_1-OH and μ_2-O (right).

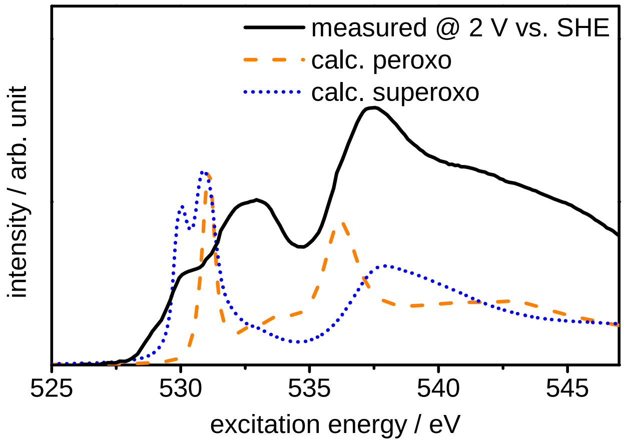

Figure S31: O K-edges computed for iridium peroxo (dashed orange) and superoxo (dotted blue) species compared to spectrum measured during OER at 2 V vs. SHE in three-electrode cell (solid black). Neither per- nor superoxo species can account for the low excitation energy feature observed at 529 eV.

References

[1] A. Knop-Gericke, E. Kleimenov, M. Hävecker, R. Blume, D. Teschner, S. Zafeiratos, R. Schlögl, V. I. Bukhtiyarov, V. V. Kaichev, I. P. Prosvirin, A. I. Nizovskii, H. Bluhm, A. Barinov, P. Dudin and M. Kiskinova, in *Advances in Catalysis*, ed. B. C. Gates and H. Knözinger, Academic Press, 2009, vol. 52, pp. 213 – 272.

[2] M. Salmeron and R. Schlögl, *Surf. Sci. Rep.*, 2008, **63**, 169 – 199.

[3] S. Tanuma, C. J. Powell and D. R. Penn, *Surf. Interface Anal.*, 1994, **21**, 165–176.

[4] M. Hävecker, M. Cavalleri, R. Herbert, R. Follath, A. Knop-Gericke, C. Hess, K. Hermann and R. Schlögl, *Phys. Status Solidi B*, 2009, **246**, 1459–1469.

[5] F. de Groot and A. Kotani, *Core level spectroscopy of solids*, CRC press, 2008.

[6] R. Arrigo, M. Hävecker, M. E. Schuster, C. Ranjan, E. Stotz, A. Knop-Gericke and R. Schlögl, *Angew. Chem. Int. Ed.*, 2013, **52**, 11660–11664.

[7] H. Bluhm, D. F. Ogletree, C. S. Fadley, Z. Hussain and M. Salmeron, *J. Phys.: Condens. Matter*, 2002, **14**, L227.

[8] V. Pfeifer, T. E. Jones, J. J. Velasco Vélez, C. Massué, M. T. Greiner, R. Arrigo, D. Teschner, F. Girgsdies, M. Scherzer, J. Allan, M. Hashagen, G. Weinberg, S. Piccinin, M. Hävecker, A. Knop-Gericke and R. Schlögl, *Phys. Chem. Chem. Phys.*, 2016, **18**, 2292–2296.

[9] V. Pfeifer, T. E. Jones, J. J. Velasco Vélez, C. Massué, R. Arrigo, D. Teschner, F. Girgsdies, M. Scherzer, M. T. Greiner, J. Allan, M. Hashagen, G. Weinberg, S. Piccinin, M. Hävecker, A. Knop-Gericke and R. Schlögl, *Surf. Interface Anal.*, 2016, **48**, 261–273.

[10] P. Giannozzi, S. Baroni, N. Bonini, M. Calandra, R. Car, C. Cavazzoni, D. Ceresoli, G. L. Chiarotti, M. Cococcioni, I. Dabo, A. Dal Corso, S. de Gironcoli, S. Fabris, G. Fratesi, R. Gebauer, U. Gerstmann, C. Gougoussis, A. Kokalj, M. Lazzeri, L. Martin-Samos, N. Marzari, F. Mauri, R. Mazzarello, S. Paolini, A. Pasquarello, L. Paulatto, C. Sbraccia, S. Scandolo, G. Sclauzero, A. P. Seitsonen, A. Smogunov, P. Umari and R. M. Wentzcovitch, *J. Phys.: Condens. Matter*, 2009, **21**, 395502.

[11] J. P. Perdew, K. Burke and M. Ernzerhof, *Phys. Rev. Lett.*, 1996, **77**, 3865–3868.

[12] A. Dal Corso, *Comp. Mater. Sci.*, 2014, **95**, 337 – 350.

[13] C. Gougoussis, M. Calandra, A. P. Seitsonen and F. Mauri, *Phys. Rev. B*, 2009, **80**, 075102.

[14] J. Rossmeisl, Z. W. Qu, H. Zhu, G. J. Kroes and J. K. Nørskov, *J. Electroanal. Chem.*, 2007, **607**, 83–89.
